# *N*′-Phenylacetohydrazide Derivatives as Potent Ebola Virus Entry Inhibitors with an Improved Pharmacokinetic Profile

**DOI:** 10.1021/acs.jmedchem.2c01785

**Published:** 2023-04-06

**Authors:** Alfonso Garcia-Rubia, Fátima Lasala, Tiziana Ginex, Marcos Morales-Tenorio, Catherine Olal, Michelle Heung, Paola Oquist, Inmaculada Galindo, Miguel Ángel Cuesta-Geijo, José M. Casasnovas, Nuria E. Campillo, Ángeles Canales, Covadonga Alonso, Ana Martínez, César Muñoz-Fontela, Rafael Delgado, Carmen Gil

**Affiliations:** † Centro de Investigaciones Biológicas Margarita Salas (CIB-CSIC), Madrid 28040, Spain; ‡ Instituto de Investigación Hospital 12 de Octubre, Madrid 28041, Spain; § Bernhard Nocht Institute for Tropical Medicine, Hamburg 20359, Germany; ∥ Facultad de Ciencias Químicas, Universidad Complutense de Madrid, Madrid 28040, Spain; ⊥ Dpt. Biotechnology, Instituto Nacional de Investigación y Tecnología Agraria y Alimentaria (INIA-CSIC), Madrid 28040, Spain; # Centro Nacional de Biotecnología (CNB-CSIC), Madrid 28049, Spain; ∇ Instituto de Ciencias Matemáticas (ICMAT-CSIC), Madrid 28049, Spain

## Abstract

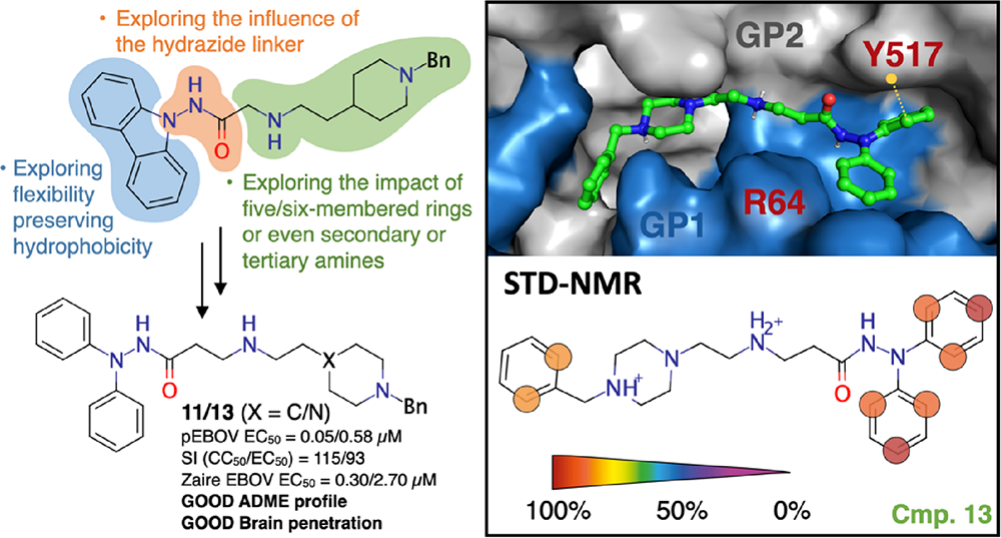

Ebola virus (EBOV) is a single-strand RNA virus belonging to the *Filoviridae* family, which has been associated to most Ebola virus disease outbreaks to date, including the West African and the North Kivu epidemics between 2013 and 2022. This unprecedented health emergency prompted the search for effective medical countermeasures. Following up on the carbazole hit identified in our previous studies, we synthetized a new series of compounds, which demonstrated to prevent EBOV infection in cells by acting as virus entry inhibitors. The *in vitro* inhibitory activity was evaluated through the screening against surrogate models based on viral pseudotypes and further confirmed using replicative EBOV. Docking and molecular dynamics simulations joined to saturation transfer difference–nuclear magnetic resonance (STD–NMR) and mutagenesis experiments to elucidate the biological target of the most potent compounds. Finally, *in vitro* metabolic stability and *in vivo* pharmacokinetic studies were performed to confirm their therapeutic potential.

## Introduction

**Figure 1:**
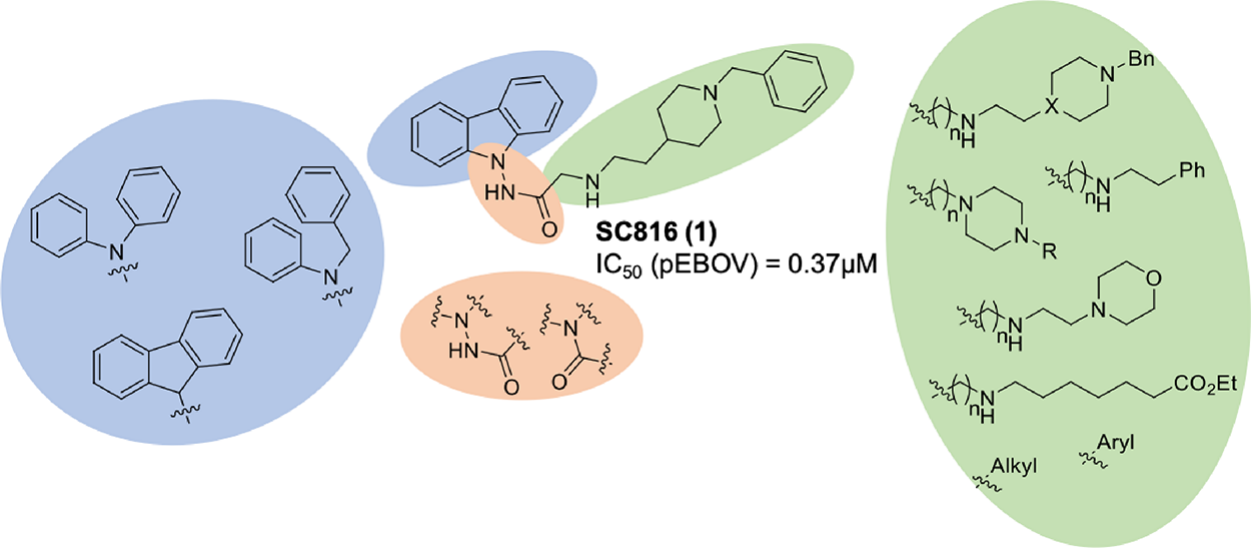
Proposed structural modifications on the carbazole hit **SC816** (**1**).

Since Ebola virus (species *Zaire ebolavirus*, EBOV) was first discovered in Africa in 1976, the virus has continued to emerge and re-emerge in this continent to the present day. The 2013–2016 West African epidemic represented the largest, longest, and deadliest ever-recorded outbreak of Ebola virus disease (EVD) followed by additional outbreaks and epidemics, such as the one that took place in North Kivu (Democratic Republic of Congo) in 2018–2020. Both epidemics were declared by the WHO as public health emergency of international concern and thus pursued the search for effective medical countermeasures and a better knowledge of the disease progression.^1,2^ EBOV is a single-strand RNA virus belonging to the *Filoviridae* family.^3^ The viral genome encodes for the nucleoprotein (NP), several viral proteins (VP24, 30, 35 and 40), the EBOV glycoproteins (GP), and the RNA-dependent RNA polymerase (L). The EBOV-GP populates the viral envelope and promotes viral entry into the host cell through the recognition of the endosomal Niemann–Pick C1 (NPC1) protein.^4,5^ In this regard, the inhibition of the NPC1/EBOV-GP interaction, either directly or indirectly (allosterically), was proposed as a therapeutic antiviral strategy to combat EBOV infection.^6^ Infection with EBOV causes EVD, which is a severe and deadly disease whose symptoms include fever, diarrhea, vomiting, bleeding, and, often, death.^7^ Management of the acute EVD was mainly based on supportive care. In 2019, the FDA approved the first EBOV vaccine, rVSV-EBOV-GP (Ervebo by Merck). It is a life and attenuated recombinant vesicular stomatitis virus (rVSV) vaccine in which the VSV envelope GP was replaced with the EBOV-GP to induce antibodies production.^8^ With regards to postexposure therapy, two biopharmaceuticals, REGN-EB3 as a mixture of three monoclonal antibodies (Inmazeb by Regeneron Pharmaceuticals), and the single monoclonal antibody, mAb114 (Ebanga by Ridgebak), were also approved by the FDA in 2020. Remarkably, both therapeutics target different epitopes in EBOV-GP.^9^ Despite the efficacy of vaccine and treatments, outbreaks continue to unpredictably occur in Africa. Considering the low/middle income of most of African countries, it is of utmost importance the development of cost-effective therapeutics.^10^ In this direction, we have focused our efforts in the development of small molecules with antiviral properties against EBOV whose main advantages would consist on the cost-effective production, easy storage, and even a potential oral administration.^11,12^ Previously, we have identified the carbazole derivative **SC816** (**1**) (Figure 1) as a promising antiviral hit, which showed antiviral activity at the low micromolar range in a lentiviral EBOV-GP-pseudotyped infection (pEBOV) assay. According to our previous data, the compound is expected to act through inhibition of the viral entry process.^13^ Based on these preliminary results, we carried out here a hit-to-lead optimization of the carbazole **SC816** (**1**) (Figure 1), aiming to evolve antivirals with a better pharmacotherapeutic profile.

Of note, molecular dynamics (MD) simulations and saturation transfer difference–nuclear magnetic resonance (STD–NMR) experiments were also performed to identify the putative binding mode of these newly synthesized antivirals in their biological target. Finally, the therapeutic potential of the most potent compounds was examined and confirmed through *in vitro* metabolic stability and *in vivo* pharmacokinetic studies.

## Results and Discussion

### Chemistry

**Figure 2:**
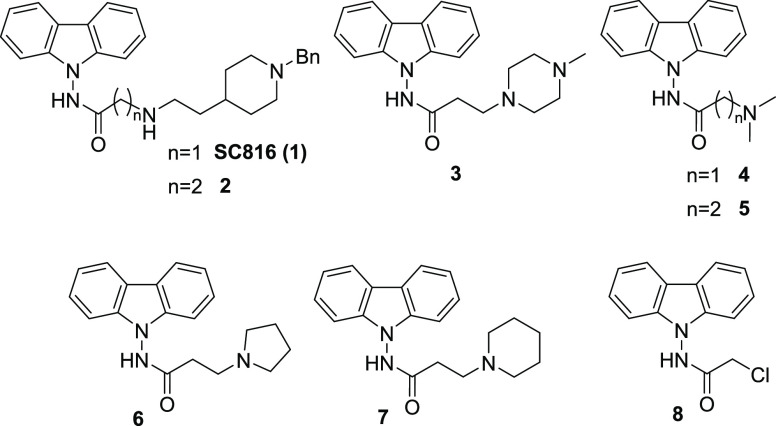
Carbazole derivatives from our in-house MBC library structurally related to the carbazole hit **1**.

Starting with our previous identified hit, the carbazole derivative **SC816** (**1**), different modifications were done to improve not only the antiviral potency but also the therapeutic window by measuring the cytotoxicity in animal cells. First of all, a similarity search based on the carbazole scaffold was carried out on our in-house chemical library, named MBC library.^14^ As a result, seven different carbazole derivatives were selected and evaluated (Figure 2). These new compounds were derivatized, functionalizing the carbazole ring with different substituents through the hydrazide linker. The length and nature of the lateral chain is variable, including five or six-membered rings or even secondary or tertiary amines (Figure 2).

**Scheme 1:**
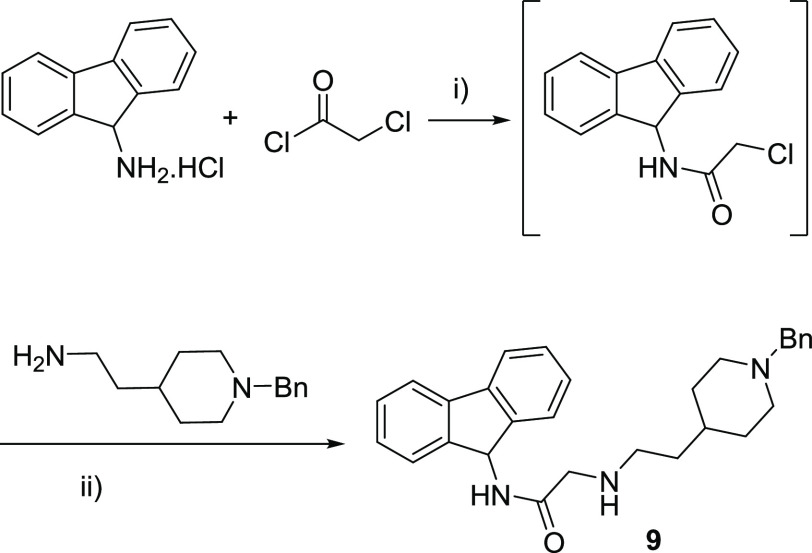
Synthesis of the Fluorene-Bearing Derivative **9** Reagents and conditions: (i) Et_3_N, CH_2_Cl_2_, rt, 16 h; (ii) Et_3_N, ACN, reflux, 16 h.

Afterward, the replacement of the carbazole ring with a fluorene was explored to assess the impact of a heterocyclic nitrogen atom on the biological activity. Accordingly, the commercial 9*H*-fluoren-9-amine hydrochloride reacted with 2-chloroacetyl chloride in dichloromethane to yield the corresponding chloride that formed subsequently fluorene derivative **9** after treatment with the appropriate amine (Scheme 1).

**Table 1:**
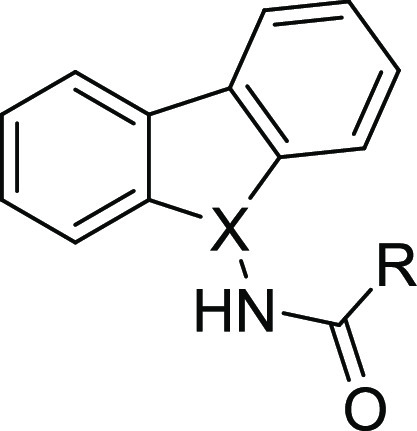

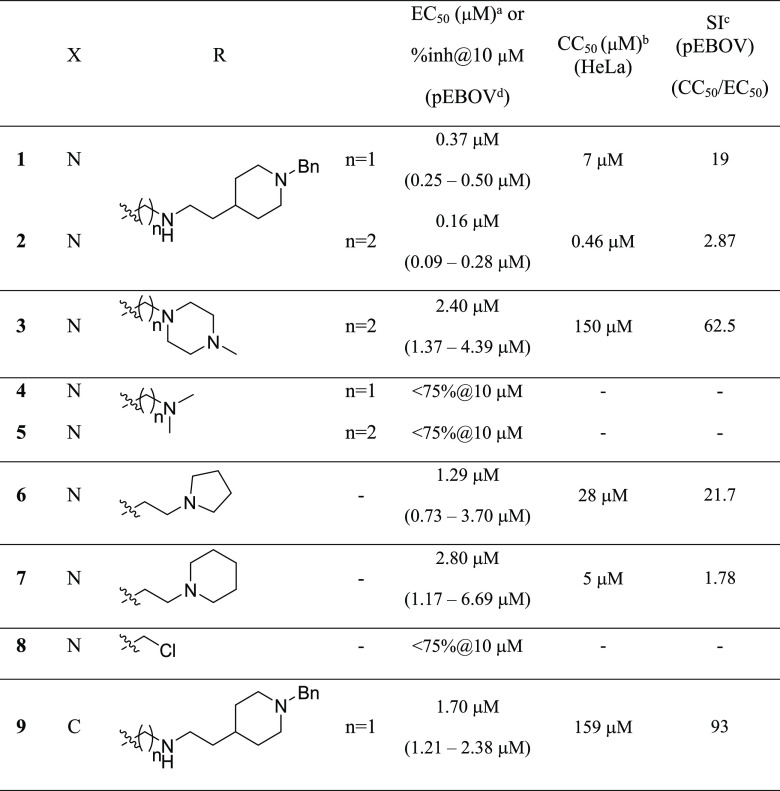
Antiviral Activity of the Carbazole and Fluorene Derivatives **1**–**9** against EBOV-GP-Pseudotype Virus (pEBOV)

a EC_50_: 50% effective concentration (with 95% confidence intervals in parentheses).

b CC_50_: 50% cytotoxic concentration.

c SI: selectivity index.

d Toremifene was used as a reference of the assay EC_50_ = 0.07 ± 0.05 μM.^15^

**Scheme 2:**
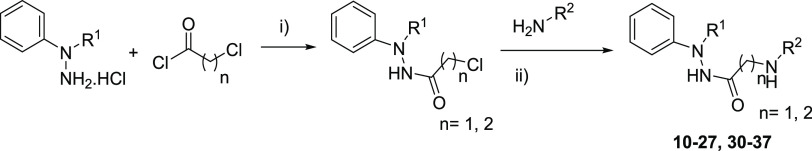
Synthesis of the *N*′,*N*′-Diphenyl and *N*′-Benzyl-*N*′-phenyl Derivatives **10**–**27** and **30**–**37** Reagents and conditions: (i) K_2_CO_3_, acetone/H_2_O (1:2), rt, 16 h; (ii) Et_3_N, ACN, reflux, 16 h.

The next step consisted in the increase of the conformational freedom of the carbazole ring by eliminating the bond between both phenyl rings. Thus, the carbazole scaffold was replaced by *N*′,*N*′-diphenyl or *N*′-benzyl-*N*′-phenyl amine units. The synthetic route to the proposed *N*′-phenylacetohydrazide derivatives used *N*′,*N*′-diphenylhydrazine hydrochloride or *N*′-benzyl-*N*′-phenylhydrazine hydrochloride and 2-chloroacetyl chloride as starting reactants. A number of derivatives (see **10**–**27** and **30**–**37** in Scheme 2 and Table 2) with a wide chemical diversity in term of lateral chains were thus synthesized using different amines.

**Table 2:**
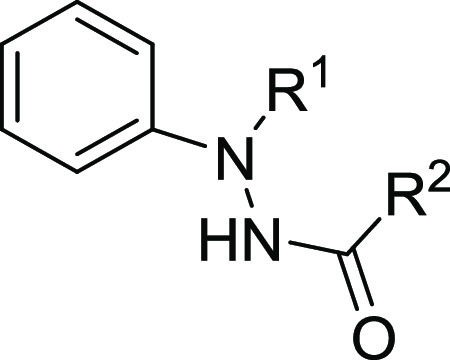

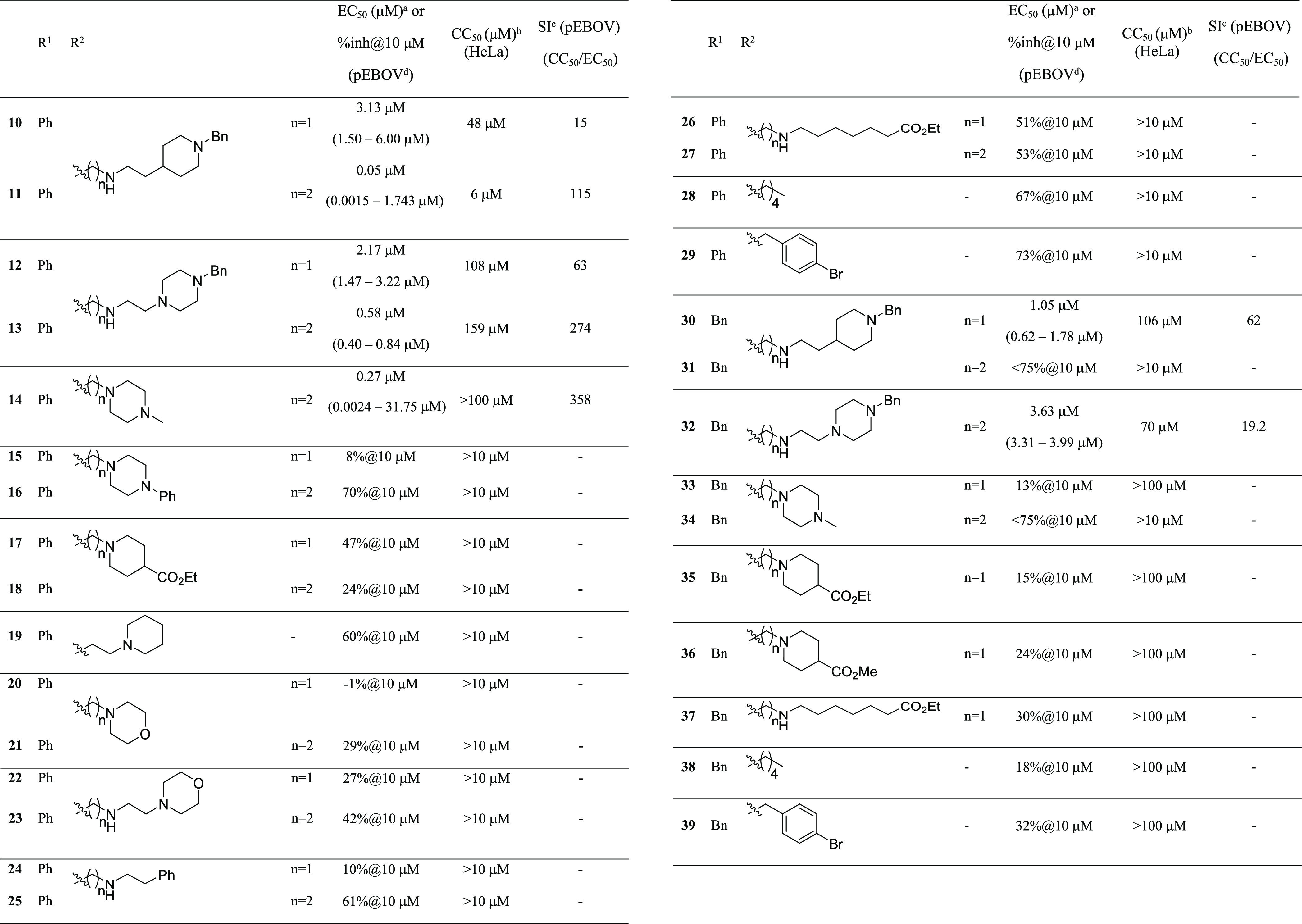
Antiviral Activity of *N*′-Phenylacetohydrazide Derivatives **10**–**39** against EBOV-GP-Pseudotype Virus (pEBOV)

a EC_50_: 50% effective concentration (with 95% confidence intervals in parentheses).

b CC_50_: 50% cytotoxic concentration.

c SI: selectivity index.

d Toremifene was used as a reference of the assay EC_50_ = 0.07 ± 0.05 μM.^15^

**Scheme 3:**
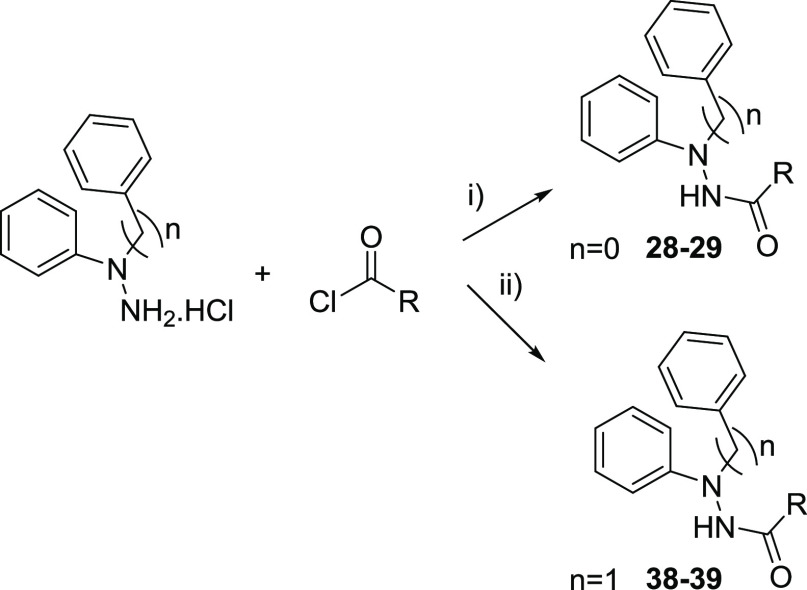
Synthesis of the *N*′,*N*′-Diphenyl and *N*′-Benzyl-*N*′-phenyl derivatives **28**–**29** and **38**–**39**. Reagents and conditions: (i) Et_3_N, THF, rt, 16 h; (ii) DIPEA, ACN, 0 °C to rt, 16 h.

Additionally, reaction of the *N*′,*N*′-diphenylhydrazine hydrochloride or *N*′-benzyl-*N*′-phenylhydrazine hydrochloride with pentanoyl chloride and 2-(4-chlorophenyl)acetyl chloride led to the **28**–**29** and **38**–**39** derivatives with different lateral chains (Scheme 3 and Table 2).

**Scheme 4:**
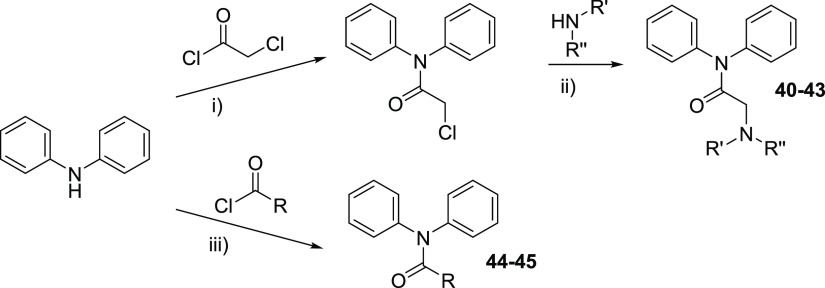
Synthesis of the *N*,*N*-Diphenylacetamide Derivatives **40**–**45** Reagents and conditions: (i) Et_3_N, CH_2_Cl_2_, rt, 16 h; (ii) Et_3_N, ACN, reflux, 16 h; (iii) DIPEA, ACN, rt, 16 h.

Finally, to check the influence of the hydrazide linker, several *N*,*N*-diphenylacetamide derivatives with an amide linker (compounds **40**–**45**) were synthesized (Scheme 4 and Table 3).

**Table 3:**
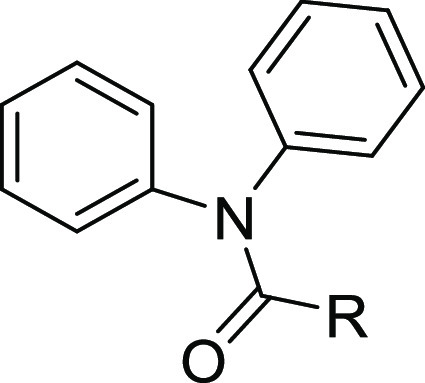

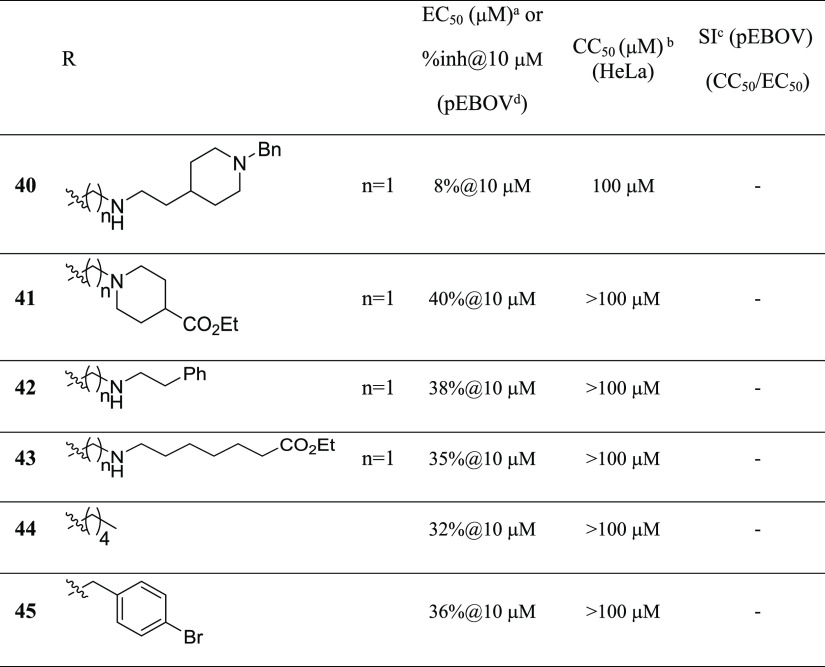
Antiviral Activity of the *N*′-Phenylacetamide Derivatives **40**–**45** against EBOV-GP-Pseudotype Virus (pEBOV)

a EC_50_: 50% effective concentration (with 95% confidence intervals in parentheses).

b CC_50_: 50% cytotoxic concentration.

c SI: selectivity index.

d Toremifene was used as a reference of the assay EC_50_ = 0.07 ± 0.05 μM.^15^

### Screening against Virus Pseudotypes and Structure–Activity Relationships (SAR)

To check the antiviral potential of all the compounds here synthesized, we decided to first use a surrogate model based on viral pseudotypes expressing on their surface, the EBOV-GP. Based on our previous findings,^13^ we hypothesized that the compounds under study would act as viral entry inhibitors; therefore, the use of pseudotypes is perfectly justified. Moreover, the fact of working under BSL-2 facilities instead of the BSL-4 required to work with authentic EBOV is a significant advantage. Collectively, this allowed to speed-up this stage of drug discovery. Noteworthy, viral pseudotypes with the vesicular stomatitis virus envelope GP (VSV-G) were used as a control for selectivity.

The carbazole hit **SC816** (**1**) was characterized by the presence of a carbazole ring connected through a hydrazide with a lateral chain bearing a (1-benzylpiperidin-4-yl)-ethyl-amine. Among the different carbazoles evaluated (Table 1), the ones with shorter chains as **4**, **5**, and **8** were found inactive, while the rest of compounds bearing longer and bulkier chains showed antiviral activity in the micromolar range. Among those with the longest chains, the carbazole hit **SC816** (**1**) and **2** showed EC_50_ values of 0.37 and 0.16 μM, respectively, with moderate selectivity indexes (19 and 2.87, respectively). It is interesting to note that, although both are structurally quite similar and exert antiviral activity in the same range, elongation of the side chain by only one carbon in compound **2** led to a more cytotoxic derivative in HeLa cells.

When the aromatic nitrogen atom of the carbazole was replaced by a carbon atom, carbazole hit **1** vs fluorene analog **9** (Table 1), a slightly decrease in the antiviral potency was observed (EC_50_ values of 0.37 vs 1.70 μM). Moreover, a significant improvement in the selectivity index (19 vs 93) was produced.

With respect to the *N*′-phenylacetohydrazide derivatives (Table 2), we decided to explore the impact of the substituent on the lateral chain by applying a mixed conservative and explorative strategy respectively consisting in the integration of (i) the best substituents found in the carbazole series and (ii) new lateral substituents of different length and nature. So, to enhance the chemical diversity, both the *N*′,*N*′-diphenyl and the *N*′-benzyl-*N*′-phenylacetohydrazide derivatives were prepared. Similarly to the carbazole series, compounds with the privileged lateral chain present in the carbazole hit **1** (**SC816**) showed antiviral activity in the micromolar range thus confirming the relevance of this substituent for activity. This was the case of the *N*′,*N*′-diphenyl derivative **10** and the *N*′-benzyl-*N*′-phenyl derivative **30** showing EC_50_ values of 3.13 and 1.05 μM, respectively. Interestingly, when this privileged chain is lengthen by one methylene unit as in carbazole **2**, we obtained the *N*′-benzyl-*N*′-phenyl **31**, which was inactive, and the *N*′,*N*′-diphenyl derivative **11**, which instead showed an EC_50_ of 0.05 μM and a good selectivity index of 115. This different behavior could be due to the chemical nature of the substituent in position R^1^ where a phenyl moiety is indeed preferred.

The *N*′,*N*′-diphenyl derivative **19** and the *N*′-benzyl-*N*′-phenyl derivative **34**, respectively implementing an *N*-(ethyl)piperidine and a methylpiperazine in R as in carbazoles **7** and **3**, were found inactive. However, as previously observed with compounds **11** and **31**, the *N*′,*N*′-diphenyl derivative **14**, which only differs from compound **34** for the presence of a phenyl group instead of a benzyl one in position R^1^, showed a good profile with an EC_50_ of 0.27 μM and a selectivity index of 358.

Piperidine to piperazine replacement in the privileged chain led to the *N*′,*N*′-diphenyl derivative **12**, while the addition of another methylene unit led to the *N*′,*N*′-diphenyl derivative **13** and the *N*′-benzyl-*N*′-phenyl derivative **32**. All them showed activities within the micromolar and submicromolar range, with the *N*′,*N*′-diphenyl derivatives having better selectivity index.

The incorporation of other substituents of variable length bearing aliphatic or aromatic moieties led to inactive compounds. Since a certain conformational freedom turned out to be favorable for the anti-EBOV activity, the replacement of the hydrazide by acetamide was also explored, leading to several *N*,*N*-diphenylacetamide derivatives (see Table 3). Unfortunately, none of these new compounds, even those that carry the privileged chain as compound **40**, were found active.

In summary, we have been able to improve significantly both potency and selectivity of the starting carbazole hit **1**. This was achieved by increasing the conformational freedom of the derivatives while keeping a similar lateral chain and hydrazide linker. Although the antiviral activity of the most potent compounds was in the same range of the carbazole hit **1** (**SC816**), an improvement of more than one order of magnitude in the selectivity index, joint to a safer profile and a better therapeutic window were achieved.

### Confirmation of the Best Candidates against EBOV

To validate our results, the carbazole hit **1** (**SC816**) and seven more compounds selected among the most active compounds identified in the surrogate system were further tested using Vero-E6 cells infected with the wild-type Zaire EBOV Mayinga 1976 strain (EBOV May). The inactive compound **33** was selected to validate the use of the pseudotype assay. As observed in Table 4, with the exception of derivative **14**, a good correlation was found among inhibitory data from pseudotype and infectious EBOV models. Remarkable similarities were found in the activities of the compounds from both assays, including the lack of activity for **33**. Interestingly, as in HeLa cells, compound **1** is less toxic in Vero cells than the similar compound **2**, although to a lesser extent. This is not surprising because it is well known that cell lines used can affect the results of toxicity studies.

**Table 4:** Antiviral Activity of Selected Compounds against Replicative EBOV^a^

compound	EC_50_ (μM)^b^	CC_50_ (μM)^c^	SI^d^ (CC_50_/EC_50_)
**1**	1.50 μM	16.56 μM	11.0
	(1.2–1.9 μM)		
**2**	0.52 μM	4.75 μM	9.1
	(0.27–1.00 μM)		
**9**	0.25 μM	33.65 μM	134.6
	(0.11–0.51 μM)		
**10**	1.90 μM	32.25 μM	16.9
	(1.31–2.78 μM)		
**11**	0.30 μM	9.47 μM	31.5
	(0.18–0.49 μM)		
**12**	7.80 μM	100.7 μM	12.9
	(5.00–11.90 μM)		
**13**	2.70 μM	35.53 μM	13.1
	(2.28–3.12 μM)		
**14**	>100 μM	>100 μM	-
**33**	>100 μM	>100 μM	-

a Favipiravir was used as a reference of the assay EC_50_ = 67 μM (56–75 μM).^16^

b EC_50_: 50% effective concentration (with 95% confidence intervals in parentheses).

c CC_50_: 50% cytotoxic concentration.

d SI: selectivity index.

Among the tested compounds, two of the most active compounds **11** with an EC_50_ of 0.30 μM and **13** with an EC_50_ of 2.70 μM were selected for further studies.

### Molecular Modeling

As we mentioned in the Introduction, EBOV-host cell attachment and its membrane fusion during the infection process are due to the bound of homotrimeric EBOV-GP with the NPC1 receptor.^17,18^ As for other fusion glycoproteins,^19^ EBOV-GP is characterized by two functional subunits (GP1 and GP2 in the cleaved and activated GP), which respectively mediate host cell recognition (by binding to the host NPC1 protein) and membrane fusion. A glycan cap generally protects the receptor binding site for NPC1, which is located in the upper part of the GP1 subunit. According to the proposed infection mechanism for EBOV,^6^ the cleavage of the glycan cap by cathepsin B/L activates the EBOV-GP, which in turn recognizes the host entry NPC1 receptor and promotes the uncoupling of the GP1-GP2 subunits, leading to membrane fusion.

In the last years, it has been reported that some FDA-approved drugs act as EBOV entry inhibitors *in vitro*.^20−22^ In these cases, the compounds bind to a hydrophobic groove shaped by the GP1 β1, β2, β3, β6, β13 strands and by the GP2 β19−β20 and α3 motifs. The binding of one of these drugs, toremifene, was reported to interfere with the fusion process, although the precise mechanism of action is still not fully understood.^20^

**Figure 3:**
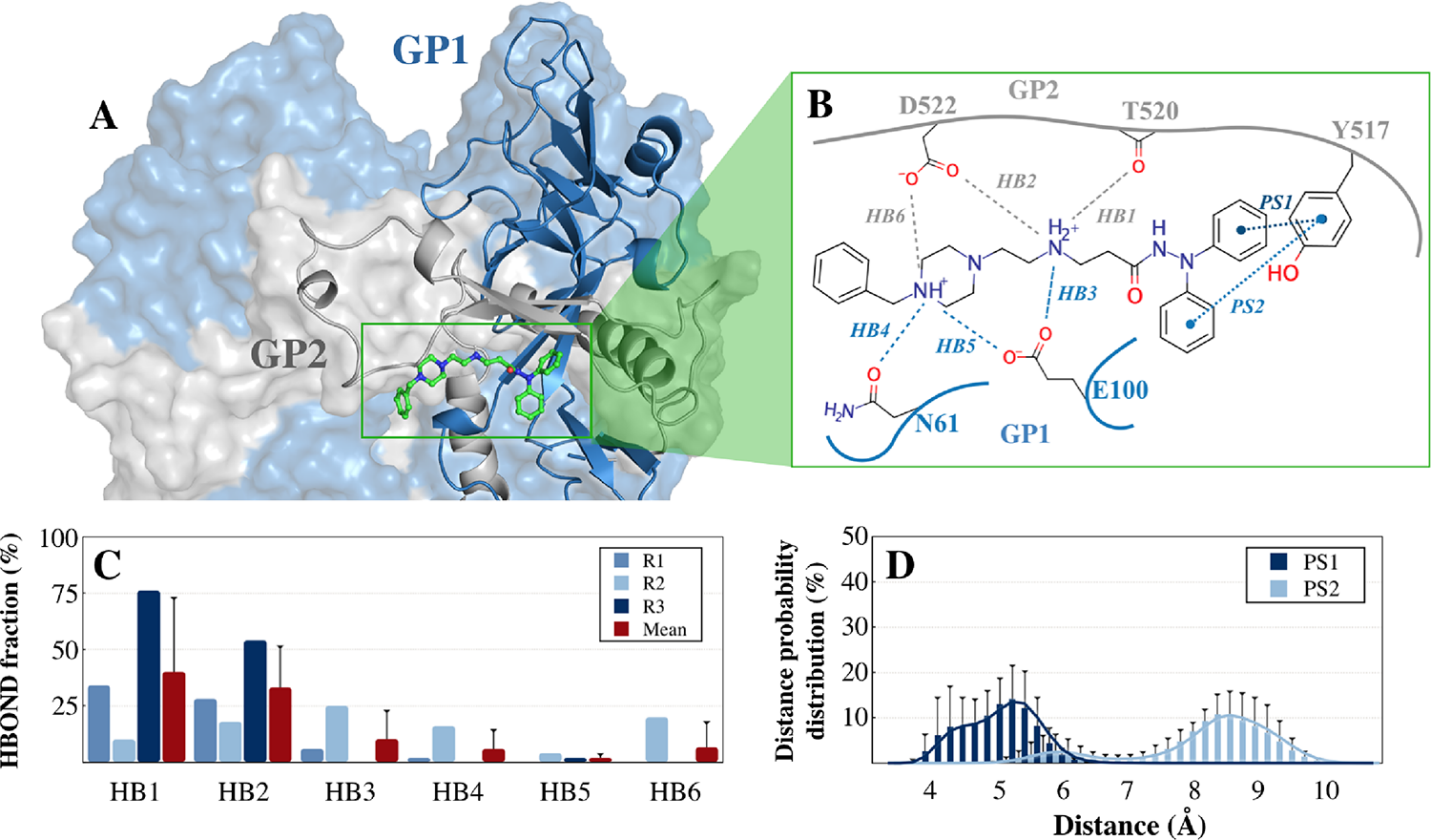
(A) Representative binding mode for compound **13** (in green sticks) in the cavity formed between the GP1 (showed as blue surface) and GP2 (showed as gray surface) of the EBOV-GP derived from 900 ns of MD simulations. (B) Cumulative representation of all the ligand–protein contacts observed along the simulated three MD replicas. (C) Per-replica (shades of blue) and global (in red) mean fraction of hydrogen-bonding (HB) interactions for the EBOV-GP-**13** complex. (D) Global ring-to-ring (PS) distance distributions between the Y517_GP2_ and two benzene rings of **13**. Standard deviation for panels C and D is reported as black bars. The X-ray structure of the EBOV-GP in complex with toremifene (PDB ID: 5JQ7)^20^ was used as a starting point for molecular modeling studies.

Docking and molecular dynamics (MD) simulations were done to elucidate the potential binding mode of compound **13**, one of the most potent antiviral inhibitors in the two antiviral assays showed above. The X-ray structure of the EBOV-GP in complex with toremifene (PDB ID: 5JQ7) was considered for molecular modeling studies.^20^ According to the fusion pH for EBOV-GP, the doubly protonated state (microspecies MS1 in Figure S1 of the Supporting Information) of **13** was docked into the binding cavity at the interface of the GP1 and GP2 of the cleaved EBOV-GP with Glide^23^ (Figure 3A).

In the EBOV-GP-toremifene complex, the ligand is mainly stabilized by a π-stacking (PS) interaction with Y517_GP2_ and also by a hydrogen-bonding (HB) interaction between the protonated aliphatic tertiary amine and E100_GP1_ (see Figure S4A of the Supporting Information). A similar binding mode was proposed for the EBOV-GP-imipramine complex^22^ with the aromatic moiety of the ligand stabilized by an edge-to-face π-stacking with Y517_GP2_ (see Figure S4B of the Supporting Information) and other hydrophobic interactions with the side chains of M548_GP2_ and L558_GP2_, although the electron density maps do not allow for unambiguous modeling of the ligand. In the docking-generated here for the EBOV-GP-**13** complex, the *N*,*N*-diphenylacetamide moiety of **13** was placed into an hydrophobic subcavity shaped by the GP2 residues Y517, M548, L558, and the GP1 residues V66 and L186. A stabilizing π-stacking interaction is proposed to be formed between one of the two phenyl rings of **13** with Y517_GP2_, while the rest of the compound is projected toward a region of the cavity with polar or charged residues such as R64_GP1_, E100_GP1_, T519_GP2_, T520_GP2_, and D522_GP2_ (see Figure 3B and Figure S4C of the Supporting Information), which participates in binding through the formation of some direct hydrogen-bonding interactions with the protonated amines of **13**. The stability of the generated binding mode was further investigated by means of 900 ns of MD simulations as a sum of three independent runs with Amber20.^24^ Analysis of the root-mean-square deviations (RMSD) values for the backbone atoms of the protein (black profiles in Figure S2A–C of the Supporting Information) and the ligand (green profiles in Figure S2A–C of the Supporting Information) confirmed the good stability of the binding mode adopted by **13** in the three simulated MD replicas. A representative snapshot of the EBOV-GP-**13** complex collected along the MD trajectory is reported in Figure 3A. A mean ring-to-ring distance of 4.8 ± 0.4 Å (PS1 in Figure 3B,D) was observed between Y517_GP2_ and one of the two phenyl rings of **13**, thus confirming the stability of the π-stacking interaction. Hydrogen-bonding interactions were also formed by the protonated secondary amine of compound **13** with the backbone oxygen of T520_GP2_ (HB1 in Figure 3C; a mean fraction of 40 ± 33%), D522_GP2_ (HB2 in Figure 3C; a mean fraction of 33 ± 19%), and/or E100_GP1_ (HB3 in Figure 3C; a mean fraction of 10 ± 13%). The protonated piperazine nitrogen was also involved in transient hydrogen bonding interactions with the amide oxygen atom of N61_GP1_ (HB4 in Figure 3C; a mean fraction of 6 ± 9%), the carboxyl moiety of E100_GP1_ (HB5 in Figure 3C; a mean fraction of 2 ± 2%), and D522_GP2_ (HB6 in Figure 3C; a mean fraction of 7 ± 11%). Indeed, a significant fluctuation in the mean values was observed for some previously reported hydrogen-bonding interactions in the EBOV-GP-toremifene complex, especially for those involving the polar or charged residues on the GP2 fusion loop. This is due to the high conformational flexibility of this region of the protein (RMSF values higher than 3 Å for the fusion loop β19−β20; Figure S3 of the Supporting Information), which is mainly unfolded and exposed to the solvent.

Comparison of the putative binding mode for compound **11**, generated after its superposition of **11** on the MD-simulated compound **13**, is shown in Figure S4C,D of the Supporting Information. Accordingly, no detrimental changes in the binding mode are expected by the replacement of the piperazine in **13** with a piperidine moiety in **11**, in line with their antiviral activity shown in Table 4.

For comparative purposes, the binding mode for the carbazole hit compound **1** (**SC816**; yellow sticks) is reported in Figure S5 of the Supporting Information. The carbazole moiety fills the hydrophobic subcavity occupied by the diphenyl moiety of **11** (in green sticks) and is involved in the π-stacking interaction with Y517_GP2_. Also, the two positively charged amines can be involved in salts bridges with E100_GP1_ and D522_GP2_ of the fusion loop. The weaker binder compound **41** (Figure S5; in magenta sticks) has been also superposed to compound **11**. As for compound **11**, the diphenyl moiety of **41** is involved in the favorable π-stacking interaction with Y517_GP2_. Here, the lower activity of compound **41** might be in part explained by the unfavorable protein–ligand interactions (i.e., charge repulsion) between the ester moiety of **41** and the negatively charged residues, E100_GP1_ and D522_GP2_. Due to a not optimal coverage of the binding site by **41**, no stabilizing interactions are formed with residues of the fusion loop β19−β20. This is expected to also contribute to weakening the stability of the binding mode.

### STD–NMR Experiments

**Figure 4:**
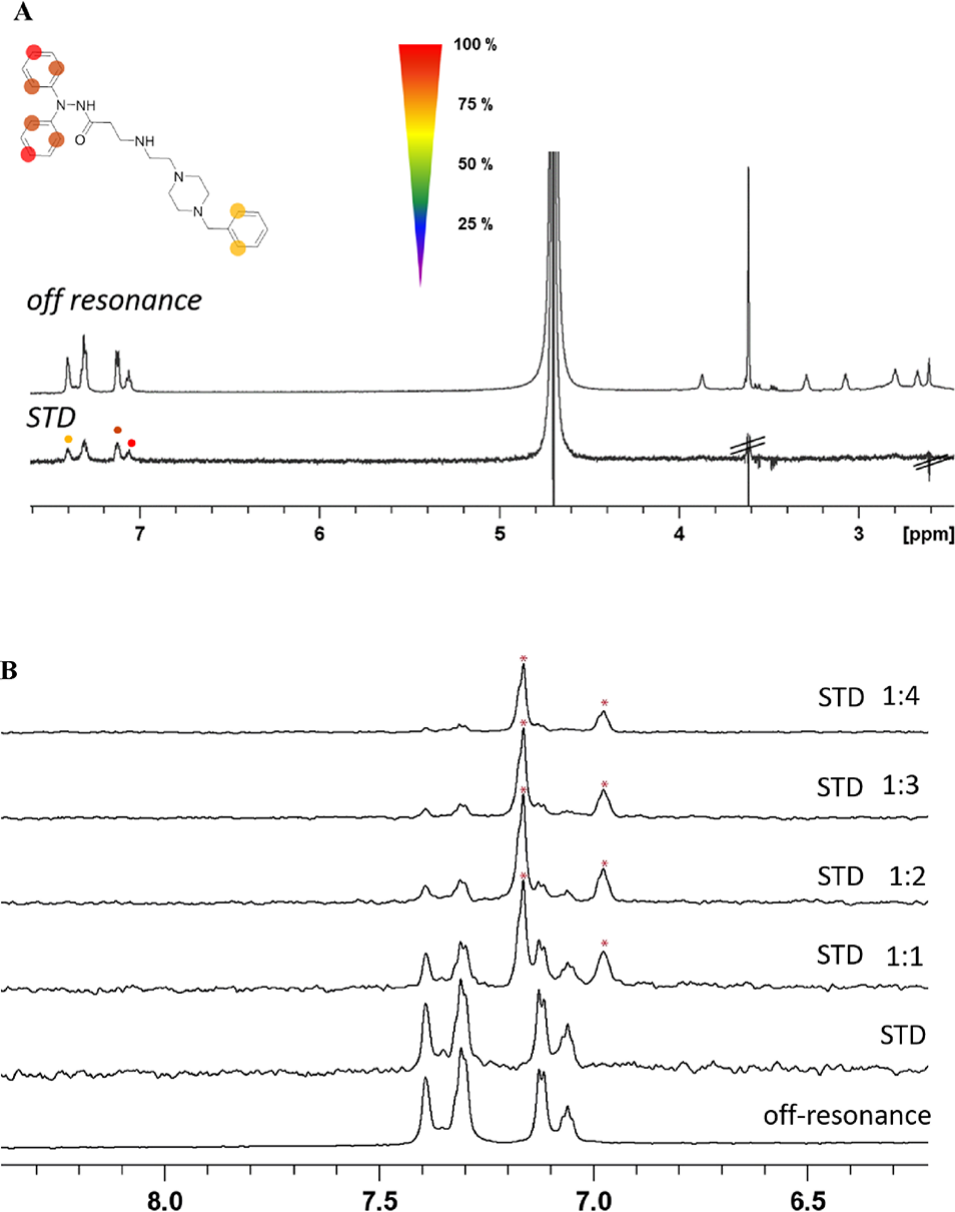
(A) Off resonance and STD spectrum of **13** in the presence of EBOV-GP protein. The signals with higher STD effects were labeled with circles. Residual buffer signals are labeled with black lines. (B) Competition experiment with increasing concentrations of imipramine. Off resonance and STD spectra of **13** in the absence of imipramine are shown as a reference (bottom spectra). Upon addition of imipramine, STD signals of **13** decrease and imipramine STD signals increase. Imipramine signals are labeled with asterisks.

To confirm the MD-proposed binding mode for the previously described new class of antivirals, saturation transfer difference–nuclear magnetic resonance (STD–NMR) experiments were acquired. This technique has proofed to be a useful method to decipher the ligand–protein interaction.^25^ Accordingly, we studied the binding of **13** to soluble EBOV-GP protein. As shown in Figure 4A, clear STD signals were detected for **13**, pointing out that the protein recognizes the compound. The STD spectrum displays signals that belongs to the three aromatic rings of the *N*′,*N*′-diphenyl derivative, indicating that they are involved in EBOV-GP recognition. These data are in agreement with the MD-derived structural model shown in Figure 3A, where the two aromatic rings of the *N*′,*N*′-diphenyl amine scaffold are located in an hydrophobic cavity contoured by GP1 and GP2. Nonetheless, STD competition experiments with imipramine, a reported EBOV inhibitor,^4^ were also carried out to evaluate whether **13** bound to the same site in EBOV-GP than imipramine.^22^ Addition of imipramine to the **13**-EBOV-GP complex led to an increase in the signal of **13**, showing that it has a weaker interaction with the target than imipramine and, therefore, the line broadening due to protein binding decreases upon addition of imipramine (Figure S6 of the Supporting Information). In addition, the displacement of **13** by imipramine induced a drastic decrease of the STD signals for **13** (see Figure 4B), thus indicating that both compounds target the same GP site.

Moreover, the STD of compound **11** has been acquired for comparison and the results are quite similar to the effects obtained for compound **13**. We detect STD effects for the three aromatic rings, and the highest values correspond to protons in the *N*,*N*′-diphenyl region (Figure S7 of the Supporting Information). In addition, the STD experiment for compound **41** that displays lower activity than **11** and **13** has been acquired. The STD spectrum of **41** shows signals, pointing out that the compound is recognized by EBOV-GP protein. This result is not surprising since STD experiment is especially a suitable technique to detect weak binders. To confirm that we are detecting a specific interaction, a competition experiment with compound **13** was also measured. In this way, addition of **13** to the sample containing **41** and EBOV-GP protein induced a decrease in the STD signals of **41** with the simultaneous appearance of the STD signals of compound **13**. Therefore, both molecules are interacting with the same binding site, although compound **41** is a weaker binder than **13** (Figure S8 of the Supporting Information). This finding correlates with the loss of activity of **41** with respect to **13**.

### Site-Specific Mutation of EBOV-GP

**Figure 5:**
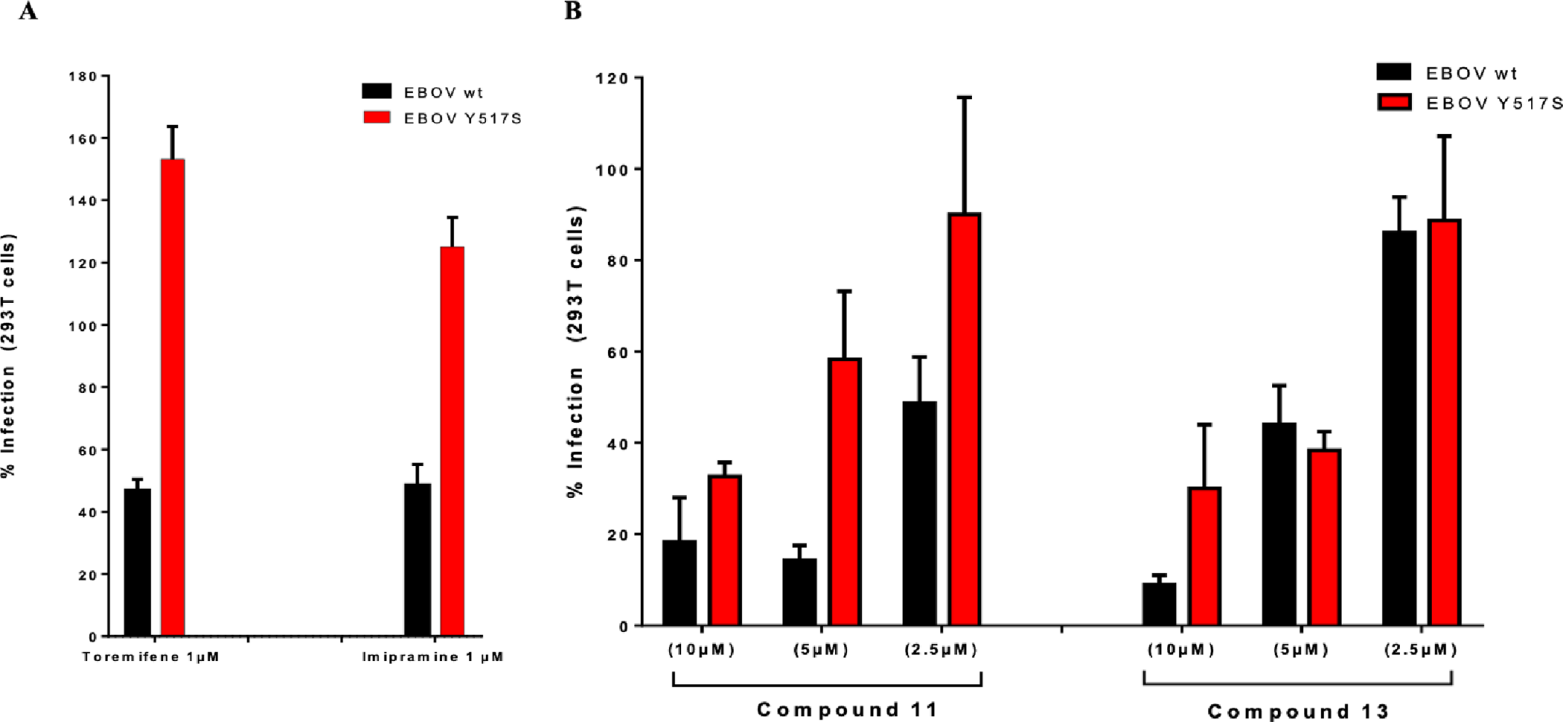
Loss of inhibitory activity of new derivatives **11** or **13** against EBOV-GP Y517S mutant-pseudotyped virus in 293T cells infected with EBOV wt or Y517S mutant-pseudotyped lentiviral particles. At 48 h, cells were lysed and assayed for luciferase expression. The percentage of infection with respect to each virus without compound, (A) in the presence of toremifene or imipramine at 1 μM or (B) in the presence of increasing concentrations of compounds **11** or **13** (2.5, 5, and 10 μM), is represented. All error bars represent s.d. from three independent experiments.

To support docking and STD–NMR studies, one GP mutant was used, Y517S. This mutant was selected because of the key role of this residue in the proposed binding mode of the here reported *N*′-phenylacetohydrazide derivatives. Moreover, previous work in the field showed how antiviral activity of toremifene significantly dropped in pseudotypes with this specific GP mutation with respect to the ones with wt GP.^26^ In the same way as toremifene,^20^ the X-ray structure of imipramine in complex with EBOV-GP also showed a key interaction with Y517_GP2_.^22^ Based on this, we first tested toremifene and imipramine in pEBOV GP Y517S to verify the lack of activity of both with respect to pEBOV wt GP (Figure 5A). After checking this fact, compounds **11** and **13** were evaluated at different concentrations. As showed in Figure 5B, while **11** showed the expected loss of activity in a dose–response manner, **13** only showed less activity at the highest concentration tested (10 μM).

These findings point to the fact that the interaction of *N*′-phenylacetohydrazide derivatives with Y517_GP2_ is key for their antiviral activity. However, more site-specific mutations in other residues as the ones described in the proposed binding mode are needed.

### Inhibition of the NPC1/EBOV-GPcl Interaction

**Figure 6:**
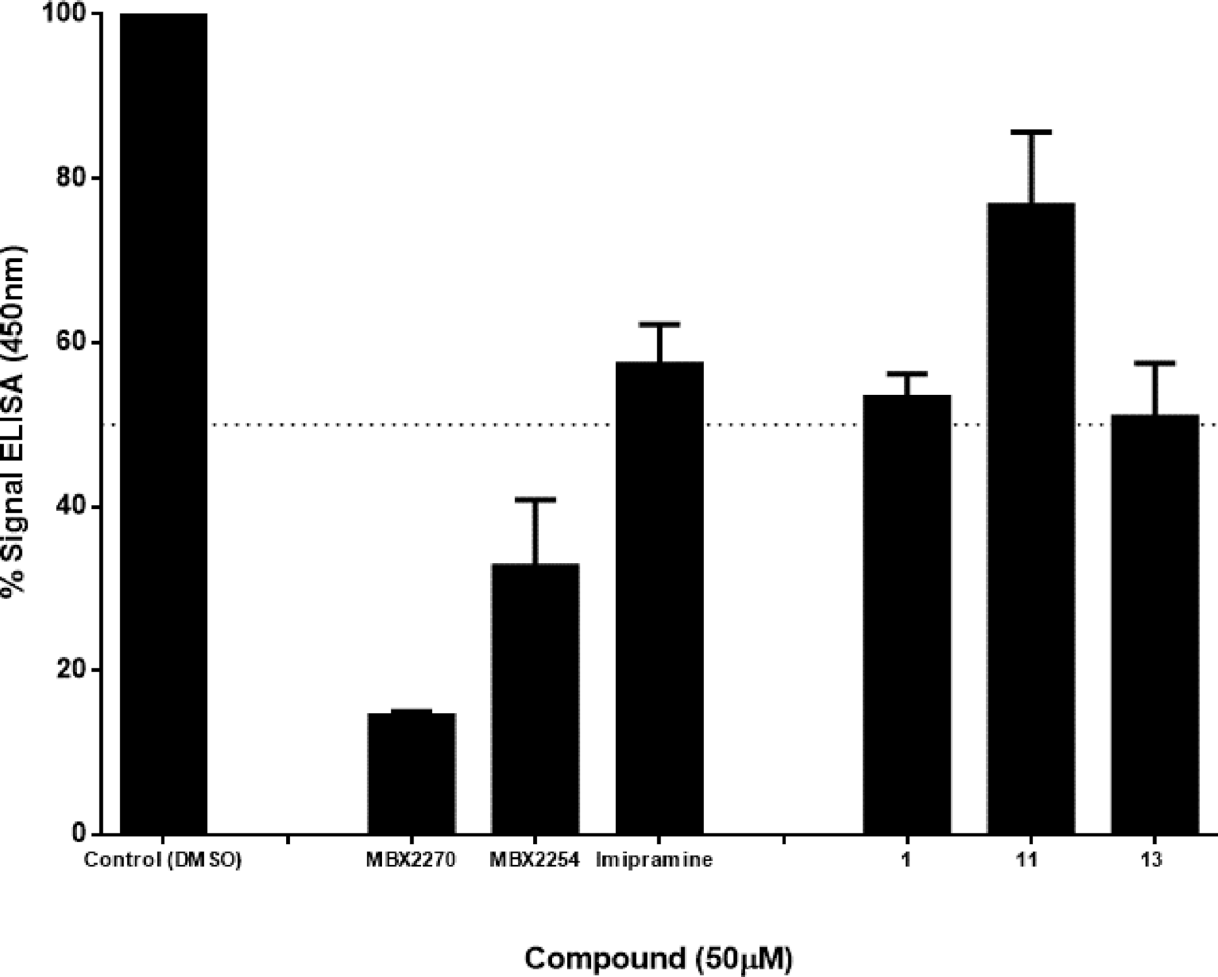
Loss of binding between EBOV-GPcl/NPC1-domain C in the presence of selected compounds measured by an ELISA. ELISA plates coated with cleaved EBOV particles were incubated with the hNPC1-domainC-flag in the presence or not (control) of compounds **1** (**SC816**), **11**, and **13**. Imipramine, MBX2270, and MBX2254 were used as a positive control. Bound domain C was detected with an anti-Flag antibody conjugated to horseradish peroxidase and the Ultra-TMB substrate. Error bars are the results of *n* = 3 experiments with the s.d.

As commented previously, the direct or indirect inhibition of the NPC1/EBOV-GP interaction is a feasible therapeutic antiviral strategy. As evidences presented here pointed to the fact that *N*′-phenylacetohydrazide derivatives bind at the GP1-GP2 interface, they could act as allosteric inhibitors of the interaction of EBOV-GP and the NPC1 receptor. To demonstrate that this new family of compounds is able to do so, we carried out an ELISA-based assay to study the effect of compounds **11** and **13** in the binding of EBOV-GP to NPC1 domain C. Previously, we showed that our hit compound **1** (**SC816**) potentially acts through inhibition of this interaction.^13^ Using the same protocol, detailed in the Experimental Section, we here showed how phenylacetohydrazide derivative **13** interferes with the NPC1/EBOV-GPcl interaction similarly than our hit **1** (**SC816**) and derivative **11** does it to a lesser extent (Figure 6). As positive controls of the assay, imipramine together with compounds MBX2270 and MBX2254 were used.^27^

### ADME Studies

To investigate the pharmacokinetic profile of selected compounds, **11** and **13**, their stability in microsomes, potential cardiotoxicity, oral absorption, and brain distribution were studied.

Both derivatives showed good metabolic stability when incubated with mouse and human microsomal fractions *in vitro* (Table 5); they showed a higher half-life and lower intrinsic clearance profile than verapamil, used as an assay control. Derivative **11** seems to have a better metabolic stability profile with mouse microsomes than **13**. By contrast, **13** showed a slightly better profile with human microsomes than **11**.

**Table 5:** *In Vitro* Microsomal Stability of Tested Compounds (**11** and **13**) in Liver Microsomes of Different Species

	metabolic stability in human liver microsomes		metabolic stability in mouse liver microsomes	
compound	*t*_1/2_ (min)	CL_int_^a^*(mL/min/mg protein)*	t_1/2_ (min)	CL_int_^a^*(mL/min/mg protein)*
**11**	45 ± 3	13 ± 1	26 ± 6	96 ± 23
**13**	53 ± 2	10.8 ± 0.3	14 ± 2	170 ± 20
verapamil	22 ± 2	26 ± 3	10 ± 1	230 ± 30

a CL_int_, intrinsic clearance.^28^

Also, the hERG (human ether-a-go-go related gene) activity of selected compounds was assayed to predict possible cardiotoxicity related to long a QT syndrome.^29^ The assessment of compound activity on the hERG channel was carried out using a FluxOR potassium assay in hERG-expressed HEK293 cells. Compounds with IC_50_ < 10 μM are classified as cardiotoxic, while higher IC_50_s are classified as noncardiotoxic.^30^ In view of the results showed in Table 6, derivatives **11** and **13** are not cardiotoxic, while the positive control, astemizole, showed and IC_50_ = 0.39 μM for the inhibition of hERG, validating the assay.

**Table 6:** hERG Potassium Channel Inhibition Induced by Tested Compounds (**11** and **13**) and a Positive Control

compound	IC_50_
**11**	16.4 μM
**13**	14.9 μM
astemizole	0.39 μM

Afterward, to study the plasma pharmacokinetic (PK) and brain distribution of **11** and **13** derivatives, a PK study in male BALB/c mice was carried out. Following a single intraperitoneal (i.p.) administration at 10 mg/kg and oral administration (p.o.) at 50 mg/kg of the compounds in male BALB/c mice, peak plasma concentrations were observed at 0.08 and 0.25 h, respectively, suggesting its rapid absorption. Brain concentrations were quantifiable up to 24 h. The brain exposures were higher than the plasma exposure, as indicated by the brain/plasma ratio (Kp) following i.p. and p.o. (Tables 7 and 8).

**Table 7:** Pharmacokinetic Parameters of Derivative **11** after Single Intraperitoneal (i.p.) Administration at 10 mg/kg and Oral Administration (p.o.) at 50 mg/kg in Male BALB/c Mice

matrix	route	dose (mg/kg)	*T*_max_^a^ (h)	*C*_max_^b^ (ng/mL)	AUC_last_^c^ (h × ng/mL)	*t*_1/2_ (h)	Brain-Kp^d^ (*C*_max_)	Brain-Kp^d^ (AUC_last_)
plasma	i.p.	10	0.08	283.42	559.47	NR^e^		
	p.o.	50	0.25	278.38	2024.42	5.87		
brain	i.p.	10	8.00	51.03	1142.02		0.18	2.04
	p.o.	50	4.00	201.45	3354.96		0.72	1.66

a *T*_max_: time to reach *C*_max_.

b *C*_max_: peak serum concentration.

c AUC_last_: area under the plasma concentration–time curve from time zero to the time of the last quantifiable concentration.

d Kp: brain/plasma ratio.

e NR: the elimination profile is not well defined.

**Table 8:** Pharmacokinetic Parameters of Derivative **13** after Single Intraperitoneal (i.p.) Administration at 10 mg/kg and Oral Administration (p.o.) at 50 mg/kg in Male BALB/c Mice

matrix	route	dose (mg/kg)	*T*_max_^a^ (h)	*C*_max_^b^ (ng/mL)	AUC_last_^c^ (h × ng/mL)	*t*_1/2_ (h)	Brain-Kp^d^ (*C*_max_)	Brain-Kp^d^ (AUC_last_)
plasma	i.p.	10	0.08	171.57	83.83	0.77		
	p.o.	50	0.25	93.62	152.76	4.36		
brain	i.p.	10	8.00	170.55	3886.74		0.99	46.36
	p.o.	50	24.00	341.33	7109.56		3.65	46.54

a *T*_max_: time to reach *C*_max_.

b *C*_max_: peak serum concentration.

c AUC_last_: area under the plasma concentration–time curve from time zero to the time of the last quantifiable concentration.

d Kp: brain/plasma ratio

While both compounds exhibited rapid absorption and good brain penetration in male BALB/c mice, we observed higher plasma levels after **11** administration. Considering data from Table 7 and the molecular weight of **11** (456 D), we can estimate a concentration of 0.62 μM on plasma after the treatment with a dose of 10 mg/Kg (i.p.). Since derivative **11** has an EC_50_ of 0.30 μM, this dose and administration route will be appropriate in a future study to test its efficacy in an animal model of the disease.

With respect to brain penetration, it is of utmost interest the fact that both compounds cross the blood–brain barrier because neurological complications such as encephalopathy were observed in survivors,^31^ and brain delivery is currently a challenge to antibody-based therapies^32^ as those approved for EVD treatment.

## Conclusions

Overall, our initial carbazole hit (**1**) was optimized leading to a novel series of *N*′-phenylacetohydrazide derivatives with antiviral activity on viral pseudotypes and improved therapeutic window. Exploration of the SAR demonstrated that the increase in the conformational freedom of the carbazole ring by eliminating the bond between both phenyl rings was favorable together with the maintenance of the hydrazide linker and a similar lateral chain than the initial hit. Moreover, the antiviral activity of this new family of compounds was confirmed in Vero cells infected with EBOV May.

Docking and molecular dynamics simulations together with STD–NMR experiments allowed to envisage the mechanism of action of these derivatives at the level of the hydrophobic cavity formed by the two subunits of EBOV-GP. In line with previous reports,^20,22^ the binding of our compounds at the interface of GP1 and GP2 near to the fusion peptide would destabilize EBOV-GP. This would prevent the attachment of EBOV-GP to the NPC1 receptor, which is necessary for viral entry and thus virus infection. Directed studies to confirm this hypothesis were followed. On one hand, mutagenesis studies pointed to the fact that the interaction of *N*′-phenylacetohydrazide derivatives with Y517_GP2_ is key for their antiviral activity. On the other, an ELISA-based assay showed the ability of this kind of antiviral compound to disrupt the NPC1/EBOV-GP interaction.

Additionally, selected *N*′,*N*′-diphenyl derivatives **11** and **13** showed good microsomal stability and low binding to the hERG channel. Both exhibited rapid absorption and good brain penetration after oral and intraperitoneal administration in mice overcoming drawbacks of current therapies.

In conclusion, this study shows *N*′,*N*′-diphenyl derivatives **11** and **13** as promising lead compounds for developing anti-EBOV drugs.

## Experimental Section

### Chemistry

All solvents were purchased from Sigma-Aldrich (anhydrous solvents), and commercially available reagents were used as received. All reactions were followed by TLC analysis (TLC plates GF254, Merck) or LC–MS (liquid chromatography mass spectrometry). Melting points were determined in a Büchi Melting Point M-560 apparatus. NMR (nuclear magnetic resonance) spectra were recorded at ambient temperature unless otherwise stated using standard pulse methods on any of the following spectrometers and signal frequencies: Bruker AV-300 (^1^H = 300 MHz, ^13^C = 75 MHz. Chemical shifts are reported in ppm and are referenced to tetramethylsilane (TMS) or the following solvent peaks: CDCl_3_ (^1^H = 7.27 ppm, ^13^C = 77.00 ppm), DMSO-*d*_6_ (^1^H = 2.50 ppm, ^13^C = 39.51 ppm). Coupling constants are quoted to the nearest 0.1 Hz, and multiplicities are given by the following abbreviations and combinations thereof: s (singlet), d (doublet), t (triplet), q (quartet), m (multiplet), and br (broad). Column chromatography was performed on prepacked silica gel columns using biotage SP4, Isolera One. High-resolution mass spectra (HRMS-ESI) were recorded on a Micromass Q-Tof Ultima hybrid quadrupole time-of-flight mass spectrometer, with analytes separated on an Agilent 1100 liquid chromatograph equipped with a Phenomenex Luna C18(2) reversed-phase column (100 mm × 2.1 mm, 3 μm packing diameter). HPLC–MS (high-pressure liquid chromatography coupled to mass spectrometry) analysis was carried out on an Agilent HPLC instrument equipped with a ZORBAX SB-C18 column (50 mm × 4.6 mm, 3.5 μM packing diameter) and Thermo Mod. Finnigan LXQ spectrometer using scan positive electrospray. Analytes were detected as a summed UV wavelength of 214, 254 and 280 nm. Two different gradient conditions were used:

Gradient I: 23 °C, 0.8 mL/min flow rate. Gradient elution with the mobile phases as (A) H_2_O containing 0.1% volume/volume (v/v) formic acid and (B) acetonitrile containing 0.1% (v/v) formic acid. Gradient conditions were initially 5% B, increasing linearly to 100% B over 3 min, remaining at 100% B for 1.45 min, and then decreasing linearly to 5% B over 0.15 min.

Gradient II: 23 °C, 0.8 mL/min flow rate. Gradient elution with the mobile phases as (A) H_2_O containing 0.1% volume/volume (v/v) formic acid and (B) acetonitrile containing 0.1% (v/v) formic acid. Gradient conditions were initially 5% B, increasing linearly to 100% B over 1 min, remaining at 100% B for 3.45 min, decreasing linearly to 15% B over 0.15 min, and finally decreasing to 5% B over 2 min.

All the final compounds are ≥95% pure by HPLC. Confirmatory HPLC traces are included in the Supporting Information.

#### Synthesis of 2-((2-(1-Benzylpiperidin-4-yl)ethyl)amino)-*N*-(9*H*-fluoren-9-yl)acetamide (**9**)

9*H*-Fluoren-9-amine (1.1 g, 5.0 mmol) and Et_3_N (0.69 mL, 10.0 mmol) were dissolved in CH_2_Cl_2_ (18 mL). Then, 2-chloroacetyl chloride (1.2 mmol) dissolved in CH_2_Cl_2_ (5 mL) was added drop by drop at 0 °C. The solution was stirred at 0 °C during 30 min and overnight at room temperature. When the reaction had finalized, the mixture was diluted with EtOAc (20 mL) and washed with HCl 2 M (3 × 20 mL), NaHCO_3(sat)_ (3 × 20 mL) and NaCl_(sat)_ (3 × 20 mL). The organic phase was dried over MgSO_4_ anh., filtered, and evaporated under reduced pressure to give 2-chloro-*N*-(9*H*-fluoren-9-yl)acetamide that was used in the next step without further purification.

The 2-chloro-*N*-(9*H*-fluoren-9-yl)acetamide (77 mg, 0.3 mmol) was dissolved in anhydrous acetonitrile (3 mL). 2-(1-Benzylpiperidin-4-yl)ethan-1-amine (65 mg, 0.3 mmol) and triethylamine (85 μL, 0.6 mmol) were then added, and the mixture was refluxed overnight. Subsequently, it was cooled to room temperature, ethyl acetate (15 mL) was added, and the solution was washed with a saturated solution of sodium chloride (10 mL × 3). The organic phase was dried, filtered, and evaporated under reduced pressure, obtaining a crude reaction that was purified in column chromatography eluting with the CH_2_Cl_2_/MeOH solvent mixture (from 99:1 to 90:10) to yield **9** (47 mg, 36%) as a white solid; m.p. 106–108 °C. ^1^H NMR (300 MHz, DMSO-*d*_6_) δ 8.25 (d, *J* = 8.6 Hz, 1H), 7.85 (d, *J* = 7.5 Hz, 2H), 7.55–7.36 (m, 4H), 7.36–7.15 (m, 7H), 6.04 (d, *J* = 8.4 Hz, 1H), 3.38 (s, 2H), 3.21 (s, 2H), 2.78–2.64 (m, 2H), 2.49 (d, *J* = 1.8 Hz, 2H), 1.76 (td, *J* = 11.5, 2.4 Hz, 2H), 1.51 (d, *J* = 11.9 Hz, 2H), 1.28 (p, *J* = 7.7, 7.1 Hz, 3H), 1.07 (td, *J* = 11.9, 3.6 Hz, 2H). ^13^C NMR (75 MHz, DMSO-*d*_6_) δ 172.7, 145.0, 140.4, 139.0, 129.0, 128.7, 128.4, 127.9, 127.0, 125.0, 120.5, 62.9, 54.2, 53.6, 52.6, 46.7, 36.6, 33.4, 32.3. HRMS (ESI) *m*/*z*: [M + H]^+^ calcd. for C_29_H_34_N_3_O 440.2697; found 440.2695. HPLC–MS (gradient I) (M + H)^+^ = 440.3, *R*_t_ = 2.11 min (95%).

### General Procedure for the Synthesis of 2-Amino-*N*′,*N*′-diphenylacetohydrazide and 3-Amino-*N*′,*N*′-diphenylpropanehydrazide Derivatives **10**–**27**

To a solution of *N*′,*N*′-diphenylhydrazine hydrochloride (2.2 g, 10 mmol) in acetone (10 mL) and water (10 mL) were added K_2_CO_3_ (2.1 g, 15 mmol) and the corresponding chloride, 2-chloroacetyl chloride (12.5 mmol, 1.0 mL), or 3-chloropropanoyl chloride (12.5 mmol, 1.2 mL). The solution was stirred overnight at room temperature. The appeared precipitate was collected by filtration, washed with water, and dried over reduced pressure to afford the title compound, 2-chloro-*N*′,*N*′-diphenylacetohydrazide or 3-chloro-*N*′,*N*′-diphenylpropanehydrazide, that was used in the next step without further purification.

Compounds **10**–**27** were then obtained from the corresponding chloride (0.3 mmol), 2-chloro-*N*′,*N*′-diphenylacetohydrazide or 3-chloro-*N*′,*N*′-diphenylpropanehydrazide, and the appropriate amine (0.3 mmol), indicated in each case.

#### 2-((2-(1-Benzylpiperidin-4-yl)ethyl)amino)-*N*′,*N*′-diphenylacetohydrazide (**10**)

2-Chloro-*N*′,*N*′-diphenylacetohydrazide (78 mg, 0.3 mmol) and 2-(1-benzylpiperidin-4-yl)ethan-1-amine (65 mg, 0.3 mmol) were used to yield **10** (44 mg, 33%) as a brown oil. ^1^H NMR (300 MHz, DMSO-*d*_6_) δ 10.89 (s, 1H), 7.36–7.23 (m, 8H), 7.17–7.02 (m, 5H), 7.02–6.88 (m, 2H), 3.53 (s, 3H), 3.51 (s, 3H), 2.80 (m, 2H), 2.67 (m, 2H), 2.00 (m, 2H), 1.66–1.53 (m, 2H), 1.43 (m, 2H), 1.26 (t, *J* = 7.2 Hz, 1H), 1.26 (m, 1H). ^13^C NMR (75 MHz, DMSO-*d*_6_) δ 169.2, 146.1, 138.0, 129.5, 129.4, 128.6, 127.5, 122.6, 119.2, 62.4, 53.3, 49.6, 46.3, 45.8, 34.8, 33.1, 31.8, 9.2. HRMS (ESI) *m*/*z*: [M + H]^+^ calcd. for C_28_H_35_N_4_O 443.2806; found 443.2792. HPLC–MS (gradient I) (M + H)^+^ = 443.3, *R*_t_ = 2.07 min (95%).

#### 3-((2-(1-Benzylpiperidin-4-yl)ethyl)amino)-*N*′,*N*′-diphenylpropanehydrazide (**11**)

3-Chloro-*N*′,*N*′-diphenylpropanehydrazide (82 mg, 0.3 mmol) and 2-(1-benzylpiperidin-4-yl)ethan-1-amine (65 mg, 0.3 mmol) were used to yield **11** (57 mg, 42%) as a yellow oil. ^1^H NMR (300 MHz, DMSO-*d*_6_) δ 10.81 (s, 1H), 7.32–7.24 (m, 9H), 7.15–7.04 (m, 4H), 7.02–6.92 (m, 2H), 3.45 (s, 2H), 3.05 (t, *J* = 7.2 Hz, 2H), 2.87–2.75 (m, 4H), 2.66 (t, *J* = 7.2 Hz, 2H), 1.89 (d, *J* = 11.0 Hz, 3H), 1.68–1.54 (m, 2H), 1.49 (m, 2H), 1.38–1.23 (m, 1H), 1.22–0.98 (m, 2H). ^13^C NMR (75 MHz, DMSO-*d*_6_) δ 169.6, 146.0, 138.7, 129.7, 129.3, 129.1, 127.2, 122.5, 119.0, 62.6, 53.3, 45.4, 43.4, 33.3, 33.1, 31.9, 30.7. HRMS (ESI) *m*/*z*: [M + H]^+^ calcd. for C_29_H_37_N_4_O 457.2962; found 457.2953. HPLC–MS (gradient I) (M + H)^+^ = 457.3, *R*_t_ = 2.11 min (95%).

#### 2-((2-(4-Benzylpiperazin-1-yl)ethyl)amino)-*N*′,*N*′-diphenylacetohydrazide (**12**)

2-Chloro-*N*′,*N*′-diphenylacetohydrazide (78 mg, 0.3 mmol) and 2-(4-benzylpiperazin-1-yl)ethan-1-amine (66 mg, 0.3 mmol) were used to yield **12** (40 mg, 30%) as a brown oil. ^1^H NMR (300 MHz, DMSO-*d*_6_) δ 10.69 (s, 1H), 7.35–7.19 (m, 9H), 7.13–7.04 (m, 5H), 7.02–6.91 (m, 2H), 3.40 (s, 2H), 3.29 (s, 2H), 2.63 (t, *J* = 6.2 Hz, 2H), 2.36 (dt, *J* = 13.6, 4.6 Hz, 10H). ^13^C NMR (75 MHz, DMSO-*d*_6_) δ 171.5, 146.1, 138.6, 129.4, 129.2, 128.5, 127.3, 122.4, 119.1, 62.5, 57.7, 53.1, 53.0, 51.2, 46.3. HRMS (ESI) *m*/*z*: [M + H]^+^ calcd. for C_27_H_34_N_5_O 444.2758; found 444.2743. HPLC–MS (gradient I) (M + H)^+^ = 444.3, *R*_t_ = 2.09 min (95%).

#### 3-((2-(4-Benzylpiperazin-1-yl)ethyl)amino)-*N*′,*N*′-diphenylpropanehydrazide (**13**)

^1^H NMR (300 MHz, DMSO-*d*_6_) δ 10.76 (s, 1H), 7.32–7.24 (m, 9H), 7.10 (dd, *J* = 7.5, 1.6 Hz, 4H), 6.98 (t, *J* = 7.3 Hz, 2H), 3.43 (s, 2H), 3.02 (t, *J* = 6.9 Hz, 3H), 2.86 (t, *J* = 6.0 Hz, 2H), 2.59 (t, *J* = 6.9 Hz, 2H), 2.50–2.30 (m, 10H). ^13^C NMR (75 MHz, DMSO-*d*_6_) δ 170.3, 146.0, 138.5, 129.7, 129.3, 129.1, 128.4, 122.4, 119.0, 62.3, 55.3, 53.0, 52.7, 44.8, 44.1, 31.5. HRMS (ESI) *m*/*z*: [M + H]^+^ calcd. for C_28_H_36_N_5_O 458.2914; found 458.2906. HPLC–MS (gradient I) (M + H)^+^ = 458.3, *R*_t_ = 2.10 min (95%).

#### 3-(4-Methylpiperazin-1-yl)-*N*′,*N*′-diphenylpropanehydrazide (**14**)

3-Chloro-*N*′,*N*′-diphenylpropanehydrazide (82 mg, 0.3 mmol) and 1-methylpiperazine (33 μL, 0.3 mmol) were used to yield **14** (44 mg, 44%) as a white solid; m.p. 176–178 °C. ^1^H NMR (300 MHz, DMSO-*d*_6_) δ 10.56 (s, 1H), 7.36–7.21 (m, 4H), 7.21–7.04 (m, 4H), 7.02–6.88 (m, 2H), 2.70–2.53 (m, 10H), 2.36 (m, 5H). ^13^C NMR (75 MHz, DMSO-*d*_6_) δ 170.9, 146.1, 129.3, 122.4, 119.1, 54.4, 53.9, 51.8, 45.1, 31.8. HRMS (ESI) *m*/*z*: [M + H]^+^ calcd. for C_20_H_27_N_4_O 339.2180; found 339.2173. HPLC–MS (gradient I) (M + H)^+^ = 339.2, *R*_t_ = 2.13 min (98%).

#### *N*′,*N*′-Diphenyl-2-(4-phenylpiperazin-1-yl)acetohydrazide (**15**)

2-Chloro-*N*′,*N*′-diphenylacetohydrazide (78 mg, 0.3 mmol) and 1-phenylpiperazine (46 μL, 0.3 mmol) were used to yield **15** (71 mg, 61%) as a white solid; m.p. 92–94 °C. ^1^H NMR (300 MHz, DMSO-*d*_6_) δ 10.59 (s, 1H), 7.29 (dd, *J* = 8.6, 7.2 Hz, 4H), 7.21 (dd, *J* = 8.7, 7.2 Hz, 2H), 7.14–7.07 (m, 4H), 7.02–6.91 (m, 4H), 6.77 (t, *J* = 7.2 Hz, 1H), 3.21–3.16 (m, 6H), 2.64 (dd, *J* = 6.1, 3.8 Hz, 4H). ^13^C NMR (75 MHz, DMSO-*d*_6_) δ 169.2, 151.3, 146.1, 129.3, 129.2, 122.3, 119.1, 119.0, 115.7, 60.0, 53.2, 48.3. HRMS (ESI) *m*/*z*: [M + H]^+^ calcd. for C_24_H_27_N_4_O 387.2180; found 387.2176. HPLC–MS (gradient I) (M + H)^+^ = 387.2, *R*_t_ = 2.82 min (95%).

#### *N*′,*N*′-Diphenyl-3-(4-phenylpiperazin-1-yl)propanehydrazide (**16**)

3-Chloro-*N*′,*N*′-diphenylpropanehydrazide (82 mg, 0.3 mmol) and 1-phenylpiperazine (46 μL, 0.3 mmol) were used to yield **16** (54 mg, 44%) as a white solid; m.p. 154–156 °C. ^1^H NMR (300 MHz, DMSO-*d*_6_) δ 10.52 (s, 1H), 7.30–7.20 (m, 6H), 7.20–7.13 (m, 4H), 7.04–6.89 (m, 4H), 6.79 (t, *J* = 7.3 Hz, 1H), 3.17 (dd, *J* = 6.4, 3.5 Hz, 4H), 2.66 (t, *J* = 6.5 Hz, 2H), 2.59 (t, *J* = 5.0 Hz, 4H), 2.39 (t, *J* = 6.5 Hz, 2H). ^13^C NMR (75 MHz, DMSO-*d*_6_) δ 170.9, 151.4, 145.9, 129.3, 129.2, 122.2, 119.2, 119.0, 115.7, 54.0, 52.9, 48.6, 31.7. HRMS (ESI) *m*/*z*: [M + H]^+^ calcd. for C_25_H_29_N_4_O 401.2336; found 401.2329. HPLC–MS (gradient I) (M + H)^+^ = 401.2, *R*_t_ = 2.72 min (95%).

#### Ethyl 1-(2-(2,2-Diphenylhydrazinyl)-2-oxoethyl)piperidine-4-carboxylate (**17**)

2-Chloro-*N*′,*N*′-diphenylacetohydrazide (78 mg, 0.3 mmol) and ethyl piperidine-4-carboxylate (47 μL, 0.3 mmol) were used to yield **17** (64 mg, 56%) as a white solid; m.p. 93–95 °C. ^1^H NMR (300 MHz, DMSO-*d*_6_) δ 10.52 (s, 1H), 7.28 (dd, *J* = 8.6, 7.2 Hz, 4H), 7.15–7.02 (m, 4H), 7.02–6.86 (m, 2H), 4.07 (q, *J* = 7.1 Hz, 2H), 3.08 (s, 2H), 2.88–2.74 (m, 2H), 2.16 (td, *J* = 11.3, 2.8 Hz, 2H), 1.86–1.57 (m, 4H), 1.18 (t, *J* = 7.1 Hz, 3H). ^13^C NMR (75 MHz, DMSO-*d*_6_) δ 174.7, 169.5, 146.1, 129.2, 122.3, 119.0, 60.4, 60.1, 53.0, 28.1, 14.4. HRMS (ESI) *m*/*z*: [M + H]^+^ calcd. for C_21_H_27_N_4_O_3_ 382.2131; found 382.2174. HPLC–MS (gradient I) (M + H)^+^ = 382.2, *R*_t_ = 2.53 min (95%).

#### Ethyl 1-(3-(2,2-Diphenylhydrazinyl)-3-oxopropyl)piperidine-4-carboxylate (**18**)

3-Chloro-*N*′,*N*′-diphenylpropanehydrazide (82 mg, 0.3 mmol) and ethyl piperidine-4-carboxylate (47 μL, 0.3 mmol) were used to yield **18** (41 mg, 35%) as a white solid; m.p. 99–101 °C. ^1^H NMR (300 MHz, DMSO-*d*_6_) δ 10.46 (s, 1H), 7.31–7.26 (m, 4H), 7.21–7.13 (m, 4H), 7.04–6.76 (m, 2H), 4.10 (q, *J* = 7.1 Hz, 2H), 2.88 (dt, *J* = 11.3, 3.8 Hz, 2H), 2.57 (t, *J* = 6.4 Hz, 2H), 2.33 (t, *J* = 6.5 Hz, 3H), 2.00 (td, *J* = 11.4, 2.5 Hz, 2H), 1.82 (dd, *J* = 13.1, 3.7 Hz, 2H), 1.71–1.56 (m, 2H), 1.21 (t, *J* = 7.1 Hz, 3H). ^13^C NMR (75 MHz, DMSO-*d*_6_) δ 175.0, 171.1, 146.0, 129.3, 122.3, 119.1, 60.2, 54.5, 52.6, 40.9, 32.1, 28.5, 14.5. HRMS (ESI) *m*/*z*: [M + H]^+^ calcd. for C_23_H_30_N_3_O_3_ 396.2282; found 396.2276. HPLC–MS (gradient I) (M + H)^+^ = 396.2, *R*_t_ = 2.53 min (99%).

#### *N*′,*N*′-Diphenyl-3-(piperidin-1-yl)propanehydrazide (**19**)

3-Chloro-*N*′,*N*′-diphenylpropanehydrazide (82 mg, 0.3 mmol) and piperidine (47 μL, 0.3 mmol) were used to yield **19** (36 mg, 37%) as a white solid; m.p. 153–155 °C. ^1^H NMR (300 MHz, DMSO-*d*_6_) δ 10.50 (s, 1H), 7.34–7.21 (m, 4H), 7.21–7.08 (m, 4H), 7.06–6.87 (m, 2H), 2.65–2.47 (m, 2H), 2.42–2.30 (m, 6H), 1.54 (p, *J* = 5.4 Hz, 4H), 1.42 (q, *J* = 6.0 Hz, 2H). ^13^C NMR (75 MHz, DMSO-*d*_6_) δ 171.0, 145.9, 129.2, 122.2, 118.9, 54.7, 54.1, 31.7, 25.9, 24.2. HRMS (ESI) *m*/*z*: [M + H]^+^ calcd. for C_20_H_26_N_3_O 324.2071; found 324.2066. HPLC–MS (gradient I) (M + H)^+^ = 324.2, *R*_t_ = 2.36 min (95%).

#### 2-Morpholino-*N*′,*N*′-diphenylacetohydrazide (**20**)

2-Chloro-*N*′,*N*′-diphenylacetohydrazide (78 mg, 0.3 mmol) and morpholine (26 μL, 0.3 mmol) were used to yield **20** (32 mg, 34%) as a colorless oil. ^1^H NMR (300 MHz, DMSO-*d*_6_) δ 10.56 (s, 1H), 7.28 (dd, *J* = 8.6, 7.2 Hz, 4H), 7.14–7.05 (m, 4H), 7.01–6.89 (m, 2H), 3.74–3.57 (m, 4H), 3.11 (s, 2H), 2.49–2.39 (m, 4H). ^13^C NMR (75 MHz, DMSO-*d*_6_) δ 169.1, 146.1, 129.3, 122.3, 119.0, 66.3, 60.5, 53.7. HRMS (ESI) *m*/*z*: [M + H]^+^ calcd. for C_18_H_22_N_3_O_2_ 312.1707; found 312.1708. HPLC–MS (gradient I) (M + H)^+^ = 312.2, *R*_t_ = 2.15 min (99%).

#### 3-Morpholino-*N*′,*N*′-diphenylpropanehydrazide (**21**)

3-Chloro-*N*′,*N*′-diphenylpropanehydrazide (82 mg, 0.3 mmol) and morpholine (26 μL, 0.3 mmol) were used to yield **21** (28 mg, 29%) as a white solid; m.p. 141–143 °C. ^1^H NMR (300 MHz, DMSO-*d*_6_) δ 10.47 (s, 1H), 7.29 (dd, *J* = 8.6, 7.1 Hz, 4H), 7.20–7.11 (m, 4H), 7.03–6.91 (m, 2H), 3.62 (t, *J* = 4.6 Hz, 4H), 2.59 (t, *J* = 6.6 Hz, 2H), 2.41 (dd, *J* = 5.9, 3.4 Hz, 4H), 2.35 (t, *J* = 6.6 Hz, 2H). ^13^C NMR (75 MHz, DMSO-*d*_6_) δ 171.0, 146.1, 129.3, 122.4, 119.1, 66.7, 54.7, 53.5, 31.6. HRMS (ESI) *m*/*z*: [M + H]^+^ calcd. for C_19_H_24_N_3_O_2_ 326.1863; found 326.1863. HPLC–MS (gradient I) (M + H)^+^ = 326.2, *R*_t_ = 2.26 min (99%).

#### 2-((2-Morpholinoethyl)amino)-*N*′,*N*′-diphenylacetohydrazide (**22**)

2-Chloro-*N*′,*N*′-diphenylacetohydrazide (78 mg, 0.3 mmol) and 2-morpholinoethan-1-amine (40 μL, 0.3 mmol) were used to yield **22** (42 mg, 40%) as a brown oil. ^1^H NMR (300 MHz, DMSO-*d*_6_) δ 10.68 (s, 1H), 7.28 (dd, *J* = 8.6, 7.2 Hz, 4H), 7.15–7.02 (m, 5H), 7.02–6.90 (m, 2H), 3.53 (t, *J* = 4.7 Hz, 4H), 3.33 (s, 2H), 2.66 (t, *J* = 6.3 Hz, 2H), 2.40 (t, *J* = 6.2 Hz, 2H), 2.33 (t, *J* = 4.8 Hz, 4H). ^13^C NMR (75 MHz, DMSO-*d*_6_) δ 171.0, 146.0, 129.3, 122.4, 119.0, 66.5, 57.6, 53.5, 50.4, 45.5. HRMS (ESI) *m*/*z*: [M + H]^+^ calcd. for C_20_H_27_N_4_O_2_ 355.2129; found 355.2125. HPLC–MS (gradient I) (M + H)^+^ = 355.2, *R*_t_ = 2.05 min (95%).

#### 3-((2-Morpholinoethyl)amino)-*N*′,*N*′-diphenylpropanehydrazide (**23**)

3-Chloro-*N*′,*N*′-diphenylpropanehydrazide (82 mg, 0.3 mmol) and 2-morpholinoethan-1-amine (40 μL, 0.3 mmol) were used to yield **23** (36 mg, 33%) as a white solid; m.p. 120–122 °C. ^1^H NMR (300 MHz, DMSO-*d*_6_) δ 10.80 (s, 1H), 7.28 (dd, *J* = 8.6, 7.2 Hz, 4H), 7.17–7.02 (m, 5H), 7.02–6.90 (m, 2H), 3.55 (t, *J* = 4.6 Hz, 4H), 3.04 (t, *J* = 6.9 Hz, 2H), 2.87 (t, *J* = 6.2 Hz, 2H), 2.61 (t, *J* = 7.0 Hz, 2H), 2.48 (d, *J* = 6.1 Hz, 2H), 2.39–2.19 (m, 4H). ^13^C NMR (75 MHz, DMSO-*d*_6_) δ 170.2, 146.0, 129.3, 122.4, 119.0, 66.4, 55.7, 53.4, 44.4, 44.1, 31.3. HRMS (ESI) *m*/*z*: [M + H]^+^ calcd. for C_21_H_29_N_4_O_2_ 369.2285; found 369.2285. HPLC–MS (gradient I) (M + H)^+^ = 369.2, *R*_t_ = 2.07 min (95%).

#### 2-(Phenethylamino)-*N*′,*N*′-diphenylacetohydrazide (**24**)

2-Chloro-*N*′,*N*′-diphenylacetohydrazide (78 mg, 0.3 mmol) and phenethylamine (38 μL, 0.3 mmol) were used to yield **24** (42 mg, 40%) as a white solid; m.p. 92–94 °C. ^1^H NMR (300 MHz, DMSO-*d*_6_) δ 10.48 (s, 1H), 7.32–7.17 (m, 9H), 7.13–7.03 (m, 4H), 7.02–6.91 (m, 2H), 3.34 (s, 1H), 3.29 (s, 2H), 2.75 (d, *J* = 1.3 Hz, 2H). ^13^C NMR (75 MHz, DMSO-*d*_6_) δ 171.1, 146.1, 140.5, 129.3, 128.9, 128.6, 126.2, 122.4, 119.0, 51.1, 51.0, 36.1. HRMS (ESI) *m*/*z*: [M + H]^+^ calcd. for C_18_H_22_N_3_O_2_ 346.1914; found 346.1916. HPLC–MS (gradient I) (M + H)^+^ = 346.2, *R*_t_ = 2.62 min (99%).

#### 3-(Phenethylamino)-*N*′,*N*′-diphenylpropanehydrazide (**25**)

3-Chloro-*N*′,*N*′-diphenylpropanehydrazide (82 mg, 0.3 mmol) and phenethylamine (38 μL, 0.3 mmol) were used to yield **25** (49 mg, 46%) as a white solid; m.p. 98–100 °C. ^1^H NMR (300 MHz, DMSO-*d*_6_) δ 10.51 (s, 1H), 7.36–7.17 (m, 9H), 7.17–7.04 (m, 5H), 7.04–6.88 (m, 2H), 2.93–2.63 (m, 6H), 2.35 (t, *J* = 6.6 Hz, 2H). ^13^C NMR (75 MHz, DMSO-*d*_6_) δ 171.3, 146.1, 140.7, 129.4, 129.0, 128.7, 126.3, 122.4, 119.1, 51.1, 45.6, 36.4, 34.4. HRMS (ESI) *m*/*z*: [M + H]^+^ calcd. for C_23_H_26_N_3_O 360.2071; found 360.2066. HPLC–MS (gradient I) (M + H)^+^ = 360.2, *R*_t_ = 2.66 min (99%).

#### Ethyl 7-((2-(2,2-Diphenylhydrazinyl)-2-oxoethyl)amino)heptanoate (**26**)

2-Chloro-*N*′,*N*′-diphenylacetohydrazide (78 mg, 0.3 mmol) and ethyl 7-aminoheptanoate hydrochloride (63 mg, 0.3 mmol) were used to yield **26** (51 mg, 43%) as a white solid; m.p. 87–89 °C. ^1^H NMR (300 MHz, DMSO-*d*_6_) δ 10.52 (d, *J* = 29.8 Hz, 1H), 7.38–7.17 (m, 4H), 7.17–7.01 (m, 4H), 7.01–6.86 (m, 2H), 4.05 (q, *J* = 7.1 Hz, 2H), 3.24 (s, 2H), 2.48 (d, *J* = 7.3 Hz, 2H), 2.27 (t, *J* = 7.4 Hz, 2H), 1.62–1.43 (m, 2H), 1.40 (d, *J* = 6.9 Hz, 2H), 1.35–1.24 (m, 4H), 1.18 (t, *J* = 7.1 Hz, 3H). ^13^C NMR (75 MHz, DMSO-*d*_6_) δ 173.3, 171.4, 146.2, 129.4, 122.4, 119.1, 60.1, 51.4, 49.5, 33.9, 29.7, 28.8, 26.9, 24.9, 14.6. HRMS (ESI) *m*/*z*: [M + H]^+^ calcd. for C_23_H_32_N_3_O_3_ 398.2438; found 398.2429. HPLC–MS (gradient I) (M + H)^+^ = 398.2, *R*_t_ = 2.72 min (97%).

#### Ethyl 7-((3-(2,2-Diphenylhydrazinyl)-3-oxopropyl)amino)heptanoate (**27**)

3-Chloro-*N*′,*N*′-diphenylpropanehydrazide (82 mg, 0.3 mmol) and ethyl 7-aminoheptanoate hydrochloride (63 mg, 0.3 mmol) were used to yield **27** (46 mg, 37%) as a white solid; m.p. 108–110 °C. ^1^H NMR (300 MHz, DMSO-*d*_6_) δ 10.82 (s, 1H), 7.33–7.23 (m, 4H), 7.16–7.05 (m, 4H), 7.03–6.95 (m, 2H), 4.05 (q, *J* = 7.1 Hz, 2H), 3.09 (t, *J* = 7.3 Hz, 2H), 2.87–2.76 (m, 2H), 2.70 (t, *J* = 7.3 Hz, 2H), 2.28 (t, *J* = 7.4 Hz, 2H), 1.67–1.43 (m, 4H), 1.33–1.25 (m, 4H), 1.18 (td, *J* = 7.1, 1.4 Hz, 3H). ^13^C NMR (75 MHz, DMSO-*d*_6_) δ 173.2, 169.6, 146.1, 129.4, 122.6, 119.2, 60.1, 47.5, 43.3, 33.8, 30.4, 28.4, 26.2, 26.2, 24.6, 14.6. HRMS (ESI) *m*/*z*: [M + H]^+^ calcd. for C_24_H_34_N_3_O_3_ 412.2595; found 412.2586. HPLC–MS (gradient I) (M + H)^+^ = 412.3, *R*_t_ = 2.11 min (95%).

### General Procedure for the Synthesis of *N*′-Benzyl-2-amino-*N*′-phenylacetohydrazide and *N*′-Benzyl-3-amino-*N*′-phenylpropanehydrazide Derivatives **30**–**37**

*N*′-Benzyl-*N*′-phenylhydrazine hydrochloride (2.3 g, 10.0 mmol) and DIPEA (4.18 mL, 30.0 mmol) were dissolved in ACN (45 mL). Then, the corresponding acyl chloride, 2-chloroacetyl chloride (12.5 mmol, 1.0 mL), or 3-chloropropanoyl chloride (12.5 mmol, 1.2 mL) dissolved in ACN (5 mL) were added drop by drop at 0 °C. The solution was stirred at 0 °C during 30 min and overnight at room temperature. When the reaction had finalized, the mixture was diluted with EtOAc (30 mL) and washed with HCl 2 M (3 × 20 mL), NaHCO_3(sat)_ (3 × 20 mL), and NaCl_(sat)_ (3 × 20 mL). The organic phase was dried over MgSO_4_ anh., filtered, and evaporated under reduced pressure, obtaining a crude reaction that was purified in column chromatography eluting with the hexane/EtOAc solvent mixture (from 95:5 to 80:20) to give *N*′-benzyl-2-chloro-*N*′-phenylacetohydrazide or *N*′-benzyl-3-chloro-*N*′-phenylpropanehydrazide.

Compounds **30**–**37** were then obtained from the corresponding chloride (0.3 mmol), *N*′-benzyl-2-chloro-*N*′-phenylacetohydrazide or *N*′-benzyl-3-chloro-*N*′-phenylpropane hydrazide, and the appropriate amine (0.3 mmol), indicated in each case.

#### *N*′-Benzyl-2-((2-(1-benzylpiperidin-4-yl)ethyl)amino)-*N*′-phenylacetohydrazide (**30**)

*N*′-Benzyl-2-chloro-*N*′-phenylacetohydrazide (82 mg, 0.3 mmol) and 2-(1-benzylpiperidin-4-yl)ethan-1-amine (65 mg, 0.3 mmol) were used to yield **30** (49 mg, 36%) as an orange oil. ^1^H NMR (300 MHz, CDCl_3_) δ 8.55 (s, 1H), 7.28–7.21 (m, 11H), 7.02–6.88 (m, 1H), 6.86–6.74 (m, 3H), 4.68 (s, 2H), 3.49 (s, 1H), 3.45 (s, 2H), 3.22 (s, 2H), 2.81 (d, *J* = 11.2 Hz, 2H), 2.45–2.32 (m, 2H), 1.87 (t, *J* = 11.2 Hz, 2H), 1.49 (d, *J* = 12.4 Hz, 3H), 1.29–1.06 (m, 5H). ^13^C NMR (75 MHz, CDCl_3_) δ 170.4, 148.7, 136.8, 129.6, 129.4, 129.2, 128.6, 128.2, 128.0, 127.5, 127.2, 119.8, 113.2, 63.2, 56.3, 52.2, 47.8, 36.7, 33.4, 32.0. HRMS (ESI) *m*/*z*: [M + H]^+^ calcd. for C_29_H_37_N_4_O 457.2962; found 457.2958. HPLC–MS (gradient I) (M + H)^+^ = 457.3, *R*_t_ = 2.07 min (95%).

#### *N*′-Benzyl-3-((2-(1-benzylpiperidin-4-yl)ethyl)amino)-*N*′-phenylpropanehydrazide (**31**)

*N*′-Benzyl-3-chloro-*N*′-phenylpropanehydrazide (87 mg, 0.3 mmol) and 2-(1-benzylpiperidin-4-yl)ethan-1-amine (65 mg, 0.3 mmol) were used to yield **31** (37 mg, 26%) as a colorless oil. ^1^H NMR (300 MHz, CDCl_3_) δ 9.64 (s, 1H), 7.33–7.03 (m, 11H), 6.95–6.65 (m, 4H), 4.65 (s, 2H), 3.39 (s, 2H), 2.76 (d, *J* = 10.9 Hz, 2H), 2.68–2.62 (m, 2H), 2.40 (t, *J* = 7.2 Hz, 2H), 2.27 (t, *J* = 5.8 Hz, 2H), 1.79 (t, *J* = 10.4 Hz, 2H), 1.53–1.43 (m, 2H), 1.27–1.08 (m, 5H). ^13^C NMR (75 MHz, CDCl_3_) δ 172.3, 149.2, 137.6, 129.7, 129.6, 128.9, 128.6, 128.5, 127.8, 127.4, 119.8, 113.3, 63.7, 56.3, 54.0, 46.9, 45.4, 36.6, 34.5, 33.9, 32.5. HRMS (ESI) *m*/*z*: [M + H]^+^ calcd. for C_30_H_39_N_4_O 471.3118; found 471.3114. HPLC–MS (gradient I) (M + H)^+^ = 471.3, *R*_t_ = 2.11 min (95%).

#### *N*′-Benzyl-3-((2-(4-benzylpiperazin-1-yl)ethyl)amino)-*N*′-phenylpropanehydrazide (**32**)

*N*′-Benzyl-3-chloro-*N*′-phenylpropanehydrazide (87 mg, 0.3 mmol) and 2-(4-benzylpiperazin-1-yl)ethan-1-amine (66 mg, 0.3 mmol) were used to yield **32** (31 mg, 22%) as a colorless oil. ^1^H NMR (300 MHz, CDCl_3_) δ 9.64 (s, 1H), 7.32–7.02 (m, 11H), 6.86–6.80 (m, 2H), 6.79–6.72 (m, 1H), 4.66 (s, 2H), 3.39 (s, 2H), 2.94–2.59 (m, 3H), 2.61–2.08 (m, 14H). ^13^C NMR (75 MHz, CDCl_3_) δ 171.8, 148.9, 137.9, 137.3, 129.2, 129.2, 128.5, 128.2, 127.4, 127.1, 119.4, 113.0, 62.9, 56.8, 56.0, 53.0, 52.9, 52.9, 52.8, 45.2, 44.89, 33.9. HRMS (ESI) *m*/*z*: [M + H]^+^ calcd. for C_29_H_37_N_5_O 472.3071; found 472.3074. HPLC–MS (gradient I) (M + H)^+^ = 472.3, *R*_t_ = 2.15 min (95%).

#### *N*′-Benzyl-2-(((4-methylpiperazin-1-yl)methyl)amino)-*N*′-phenylacetohydrazide (**33**)

*N*′-Benzyl-2-chloro-*N*′-phenylacetohydrazide (82 mg, 0.3 mmol) and 1-methylpiperazine (33 μL, 0.3 mmol) were used to yield **33** (46 mg, 46%) as a white solid; m.p. 118–120 °C. ^1^H NMR (300 MHz, CDCl_3_) δ 8.47 (s, 1H), 7.36–7.08 (m, 7H), 6.94–6.73 (m, 3H), 4.69 (s, 2H), 2.94 (s, 2H), 2.26–2.18 (m, 8H), 2.17 (s, 3H). ^13^C NMR (75 MHz, CDCl_3_) δ 168.93, 149.01, 136.70, 129.28, 128.70, 128.35, 127.65, 120.11, 113.49, 60.88, 55.92, 54.97, 53.30, 45.81. HRMS (ESI) *m*/*z*: [M + H]^+^ calcd. for C_20_H_27_N_4_O 339.2180; found 339.2176. HPLC–MS (gradient I) (M + H)^+^ = 339.2, *R*_t_ = 2.24 min (98%).

#### *N*′-Benzyl-3-(4-methylpiperazin-1-yl)-*N*′-phenylpropanehydrazide (**34**)

*N*′-Benzyl-3-chloro-*N*′-phenylpropanehydrazide (87 mg, 0.3 mmol) and 1-methylpiperazine (33 μL, 0.3 mmol) were used to yield **34** (41 mg, 35%) as a white solid; m.p. 76–78 °C. ^1^H NMR (300 MHz, CDCl_3_) δ 9.97 (s, 1H), 7.38–7.23 (m, 7H), 6.94 (dt, *J* = 7.9, 1.1 Hz, 2H), 6.87 (m, 1H), 4.78 (s, 2H), 2.72–2.25 (m, 15H).^13^C NMR (75 MHz, CDCl_3_) δ 171.50, 148.90, 137.45, 129.29, 128.64, 128.29, 127.52, 119.33, 112.73, 55.17, 54.75, 53.26, 52.02, 45.70, 31.33. HRMS (ESI) *m*/*z*: [M + H]^+^ calcd. for C_21_H_29_N_4_O 353.2336; found 353.2331. HPLC–MS (gradient I) (M + H)^+^ = 471.3, *R*_t_ = 2.21 min (99%).

#### Ethyl 1-(2-(2-Benzyl-2-phenylhydrazineyl)-2-oxoethyl)piperidine-4-carboxylate (**35**)

*N*′-Benzyl-2-chloro-*N*′-phenylacetohydrazide (82 mg, 0.3 mmol) and ethyl piperidine-4-carboxylate (47 μL, 0.3 mmol) were used to yield **35** (52 mg, 44%) as a white solid; m.p. 90–92 °C. HRMS (ESI) *m*/*z*: [M + H]^+^ calcd. for C_23_H_30_N_3_O_3_ 396.2282; found 396.2279. HPLC–MS (gradient I) (M + H)^+^ = 396.2, *R*_t_ = 2.55 min (96%).

#### Methyl 1-(2-(2-Benzyl-2-phenylhydrazineyl)-2-oxoethyl)piperidine-4-carboxylate (**36**)

*N*′-Benzyl-2-chloro-*N*′-phenylacetohydrazide (82 mg, 0.3 mmol) and methyl piperidine-4-carboxylate (41 μL, 0.3 mmol) were used to yield **36** (48 mg, 42%) as a white solid; m.p. 104–106 °C. ^1^H NMR (300 MHz, DMSO-*d*_6_) δ 9.80 (s, 1H), 7.46–7.38 (m, 2H), 7.33 (m, 2H), 7.29–7.24 (m, 1H), 7.18 (m, 2H), 6.81 (m, 2H), 6.79–6.71 (m, 1H), 4.65 (s, 2H), 3.61 (s, 3H), 2.96 (s, 2H), 2.62 (d, *J* = 11.5 Hz, 2H), 2.26 (m, 1H), 2.14–1.90 (m, 2H), 1.72 (m, 2H), 1.66–1.47 (m, 2H). ^13^C NMR (75 MHz, DMSO-*d*_6_) δ 175.3, 168.9, 149.6, 138.3, 129.2, 128.6, 128.3, 127.4, 118.8, 113.1, 60.7, 56.9, 52.8, 51.8, 40.1, 28.3. HRMS (ESI) *m*/*z*: [M + H]^+^ calcd. for C_22_H_28_N_3_O_3_ 382.2125; found 382.2121. HPLC–MS (gradient I) (M + H)^+^ = 382.2, *R*_t_ = 2.38 min (96%).

#### Ethyl 7-((2-(2-Benzyl-2-phenylhydrazineyl)-2-oxoethyl)amino)heptanoate (**37**)

*N*′-Benzyl-2-chloro-*N*′-phenylacetohydrazide (82 mg, 0.3 mmol) and ethyl 7-aminoheptanoate hydrochloride (63 mg, 0.3 mmol) were used to yield **37** (70 mg, 57%) as a colorless oil. ^1^H NMR (300 MHz, DMSO-*d*_6_) δ 10.08 (s, 1H), 7.49–7.36 (m, 2H), 7.36–7.24 (m, 3H), 7.16 (dd, *J* = 8.7, 7.2 Hz, 2H), 6.80 (d, *J* = 8.3 Hz, 2H), 6.73 (d, *J* = 7.1 Hz, 1H), 4.66 (s, 2H), 4.05 (q, *J* = 7.1 Hz, 2H), 3.15 (s, 2H), 2.27 (m, 4H), 1.57–1.40 (m, 2H), 1.31 (d, *J* = 7.1 Hz, 2H), 1.25–1.07 (m, 4H), 1.18 (t, *J* = 7.1 Hz, 3H). ^13^C NMR (75 MHz, DMSO-*d*_6_) δ 173.2, 169.7, 149.3, 138.3, 129.1, 128.5, 128.0, 127.3, 118.7, 112.8, 60.0, 57.8, 56.8, 55.3, 33.8, 28.8, 27.0, 26.6, 24.8, 14.5. HRMS (ESI) *m*/*z*: [M + H]^+^ calcd. for C_24_H_33_N_3_O_3_ 412.2595; found 412.2583. HPLC–MS (gradient I) (M + H)^+^ = 412.3, *R*_t_ = 2.51 min (95%).

### General Procedure for the Synthesis of *N*′,*N*′-Diphenylhydrazide Derivatives **28** and **29**

1,1-Diphenylhydrazine hydrochloride (220.0 mg, 1.0 mmol) and Et_3_N (0.28 mL, 2.0 mmol) were dissolved in ACN (9 mL). Then, the corresponding acetyl chloride (1.2 mmol, indicated in each case) dissolved in ACN (5 mL) was added drop by drop at 0 °C. The solution was stirred overnight at room temperature. When the reaction had finalized, the mixture was diluted with EtOAc (20 mL) and washed with HCl 2 M (3 × 20 mL), NaHCO_3(sat)_ (3 × 20 mL), and NaCl_(sat)_ (3 × 20 mL). The organic phase was dried over MgSO_4_ anh., filtered, and evaporated under reduced pressure. The crude reaction was purified in column chromatography eluting with the EtOAc/hexane solvent mixture (from 95:5 to 90:10).

#### *N*′,*N*′-Diphenylhexanehydrazide (**28**)

1,1-Diphenylhydrazine hydrochloride (220.0 mg, 1.0 mmol) and hexanoyl chloride (168 μL, 1.2 mmol) were used to yield **28** (195 mg, 69%) as a white solid; m.p. 153–155 °C. ^1^H NMR (300 MHz, DMSO-*d*_6_) δ 10.47 (s, 1H), 7.28 (dd, *J* = 8.6, 7.2 Hz, 4H), 7.14–7.02 (m, 5H), 7.02–6.87 (m, 1H), 2.20 (t, *J* = 7.3 Hz, 2H), 1.57 (t, *J* = 7.2 Hz, 2H), 1.35–1.17 (m, 4H), 0.87 (t, *J* = 6.8 Hz, 3H). ^13^C NMR (75 MHz, DMSO-*d*_6_) δ 172.0, 146.1, 129.3, 122.3, 118.9, 33.4, 31.1 24.9, 22.1, 14.2. HRMS (ESI) *m*/*z*: [M + H]^+^ calcd. for C_18_H_23_N_2_O 283.1805; found 283.1795. HPLC–MS (gradient II) (M + H)^+^ = 283.2, *R*_t_ = 1.86 min (98%).

#### 2-(4-Bromophenyl)-*N*′,*N*′-diphenylacetohydrazide (**29**)

1,1-Diphenylhydrazine hydrochloride (220.0 mg, 1.0 mmol) and 2-(4-bromophenyl)acetyl chloride (178 μL, 1.2 mmol) were used to yield **29** (213 mg, 56%) as a white solid; m.p. 184–186 °C. ^1^H NMR (300 MHz, DMSO-*d*_6_) δ 10.81 (s, 1H), 7.65–7.51 (m, 2H), 7.38–7.21 (m, 6H), 7.12–7.06 (m, 4H), 3.60 (s, 2H). ^13^C NMR (75 MHz, DMSO-*d*_6_) δ 169.4, 145.9, 135.1, 131.7, 131.5, 129.3, 122.5, 118.9, 39.8. HRMS (ESI) *m*/*z*: [M + H]^+^ calcd. for C_20_H_17_BrN_2_O 381.0597; found 381.0595. HPLC–MS (gradient II) (M + H)^+^ = 381.1, *R*_t_ = 2.32 min (98%).

### General Procedure for the Synthesis of *N*′-Benzyl-*N*′-phenylhydrazide Derivatives **38** and **39**

*N*′-Benzyl-*N*′-phenylhydrazine hydrochloride (234.7 mg, 1.0 mmol) and DIPEA (0.418 mL, 3.0 mmol) were dissolved in ACN (9 mL). Then, the corresponding acyl chloride (1.2 mmol, indicated in each case) dissolved in ACN (5 mL) was added drop by drop at 0 °C. The solution was stirred at 0 °C during 30 min and overnight at room temperature. When the reaction had finalized, the mixture was diluted with EtOAc (20 mL) and washed with HCl 2 M (3 × 20 mL), NaHCO_3(sat)_ (3 × 20 mL), and NaCl_(sat)_ (3 × 20 mL). The organic phase was dried over MgSO_4_ anh., filtered, and evaporated under reduced pressure, obtaining a crude reaction that was purified in column chromatography eluting with the CH_2_Cl_2_/MeOH solvent mixture (from 99:1 to 90:10).

#### *N*′-Benzyl-*N*′-phenylhexanehydrazide (**38**)

*N*′-Benzyl-*N*′-phenylhydrazine hydrochloride (234.7 mg, 1.0 mmol) and hexanoyl chloride (168 μL, 1.2 mmol) were used to yield **38** (180 mg, 61%) as a white solid; m.p. 77–79 °C. ^1^H NMR (300 MHz, DMSO-*d*_6_) δ 9.93 (s, 1H), 7.42–7.37 (m, 2H), 7.34 (m, 2H), 7.30–7.24 (m, 1H), 7.17 (m, 2H), 6.76 (m, 2H), 6.74–6.69 (m, 1H), 4.64 (s, 2H), 2.10 (t, *J* = 7.3 Hz, 2H), 1.58–1.39 (m, 2H), 1.24 (m, 4H), 0.86 (t, *J* = 6.9 Hz, 3H). ^13^C NMR (75 MHz, DMSO-*d*_6_) δ 171.8, 149.4, 138.5, 129.2, 128.7, 128.1, 127.4, 118.5, 112.6, 56.6, 33.5, 31.2, 25.1, 22.2, 14.3. HRMS (ESI) *m*/*z*: [M + H]^+^ calcd. for C_19_H_25_N_2_O 297.1962; found 297.1955. HPLC–MS (gradient II) (M + H)^+^ = 297.2, *R*_t_ = 2.33 min (98%).

#### *N*′-Benzyl-2-(4-bromophenyl)-*N*′-phenylacetohydrazide (**39**)

*N*′-Benzyl-*N*′-phenylhydrazine hydrochloride (234.7 mg, 1.0 mmol) and 2-(4-bromophenyl)acetyl chloride (178 μL, 1.2 mmol) were used to yield **39** (260 mg, 71%) as a white solid; m.p. 140–142 °C. ^1^H NMR (300 MHz, DMSO-*d*_6_) δ 10.21 (s, 1H), 7.55–7.44 (m, 2H), 7.40–7.25 (m, 5H), 7.23–7.10 (m, 4H), 6.86–6.57 (m, 3H), 4.63 (s, 2H), 3.46 (s, 2H). ^13^C NMR (75 MHz, DMSO-*d*_6_) δ 169.3, 149.2, 138.3, 135.5, 131.7, 131.5, 129.2, 128.7, 128.2, 127.5, 120.1, 118.8, 112.7, 56.5, 40.2. HRMS (ESI) *m*/*z*: [M + H]^+^ calcd. for C_21_H_20_BrN_2_O 395.0754; found 395.0741. HPLC–MS (gradient II) (M + H)^+^ = 395.1, *R*_t_ = 2.35 min (95%).

### General Procedure for the Synthesis of 2-Amino-*N*,*N*-diphenylacetamide Derivatives **40**–**43**

Diphenylamine (846.0 mg, 1.0 mmol) and Et_3_N (2.1 mL, 15.0 mmol) were dissolved in CH_2_Cl_2_ (18 mL). Then, 2-chloroacetyl chloride (1.2 mmol) dissolved in CH_2_Cl_2_ (5 mL) was added drop by drop at 0 °C. The solution was stirred at 0 °C during 30 min and overnight at room temperature. When the reaction had finalized, the mixture was diluted with EtOAc (20 mL) and washed with HCl 2 M (3 × 20 mL), NaHCO_3_ (3 × 20 mL), and NaCl_(sat)_ (3 × 20 mL). The organic phase was dried over MgSO_4_ anh., filtered, evaporated under reduced pressure, and used without further purification.

Compounds **40**–**43** were then obtained from 2-chloro-*N*,*N*-diphenylacetamide (0.3 mmol) and the appropriate amine (0.3 mmol), indicated in each case.

#### 2-((2-(1-Benzylpiperidin-4-yl)ethyl)amino)-*N*,*N*-diphenylacetamide (**40**)

2-Chloro-*N*,*N*-diphenylacetamide (74 mg, 0.3 mmol) and 2-(1-benzylpiperidin-4-yl)ethan-1-amine (65 mg, 0.3 mmol) were used to yield **40** (46 mg, 36%) as a colorless oil. ^1^H NMR (300 MHz, DMSO-*d*_6_) δ 7.61–6.98 (m, 13H), 3.40 (s, 2H), 3.26 (s, 4H), 2.72 (d, *J* = 11.3 Hz, 2H), 2.61 (t, *J* = 6.9 Hz, 2H), 1.83 (t, *J* = 10.5 Hz, 2H), 1.51 (d, *J* = 11.0 Hz, 2H), 1.20–0.94 (m, 5H). ^13^C NMR (75 MHz, DMSO-*d*_6_) δ 170.3, 142.8, 139.0, 129.5, 129.0, 128.4, 127.1, 62.8, 55.8, 53.6, 51.6, 34.5, 33.3, 32.3. HRMS (ESI) *m*/*z*: [M + Na]^+^ calcd. for C_28_H_33_N_3_ONa 450.2516; found 450.2522. HPLC–MS (gradient I) (M + H)^+^ = 428.3, *R*_t_ = 2.64 min (95%).

#### Ethyl 1-(2-(Diphenylamino)-2-oxoethyl)piperidine-4-carboxylate (**41**)

2-Chloro-*N*,*N*-diphenylacetamide (74 mg, 0.3 mmol) and ethyl piperidine-4-carboxylate (47 μL, 0.3 mmol) were used to yield **41** (40 mg, 37%) as a colorless oil. ^1^H NMR (300 MHz, DMSO-*d*_6_) δ 7.59–7.13 (m, 10H), 4.04 (q, *J* = 7.1 Hz, 2H), 3.04 (s, 2H), 2.81–2.58 (m, 2H), 2.29–2.15 (m, 1H), 2.15–1.93 (m, 2H), 1.84–1.59 (m, 2H), 1.59–1.32 (m, 2H), 1.17 (t, *J* = 7.1 Hz, 3H). ^13^C NMR (75 MHz, DMSO-*d*_6_) δ 174.7, 169.4, 143.2, 129.6, 128.0, 60.6, 60.2, 52.3, 40.3, 28.4, 14.5. HRMS (ESI) *m*/*z*: [M + H]^+^ calcd. for C_22_H_27_N_2_O_3_ 367.2016; found 367.2024. HPLC–MS (gradient I) (M + H)^+^ = 367.2, *R*_t_ = 2.44 min (95%).

#### 2-(Phenethylamino)-*N*,*N*-diphenylacetamide (**42**)

2-Chloro-*N*,*N*-diphenylacetamide (74 mg, 0.3 mmol) and 2-phenethylamine (68 μL, 0.3 mmol) were used to yield **42** (57 mg, 58%) as a colorless oil. ^1^H NMR (300 MHz, DMSO-*d*_6_) δ 7.54–7.05 (m, 15H), 3.19 (s, 2H), 2.81–2.57 (m, 4H). ^13^C NMR (75 MHz, DMSO-*d*_6_) δ 170.8, 140.5, 129.7, 128.9, 128.5, 126.2, 51.6, 50.6, 36.1. HRMS (ESI) *m*/*z*: [M + H]^+^ calcd. for C_22_H_23_N_2_O 331.1805; found 331.1804. HPLC–MS (gradient I) (M + H)^+^ = 331.2, *R*_t_ = 2.60 min (95%).

#### Ethyl 7-((2-(Diphenylamino)-2-oxoethyl)amino)heptanoate (**43**)

2-Chloro-*N*,*N*-diphenylacetamide (74 mg, 0.3 mmol) and ethyl 7-aminoheptanoate hydrochloride (63 mg, 0.3 mmol) were used to yield **43** (69 mg, 60%) as a colorless oil. ^1^H NMR (300 MHz, DMSO-*d*_6_) δ 7.54–7.23 (m, 10H), 4.18–3.93 (m, 2H), 3.18 (s, 2H), 2.51–2.38 (m, 2H), 2.34–2.16 (m, 2H), 1.59–1.41 (m, 2H), 1.41–1.02 (m, 9H). ^13^C NMR (75 MHz, DMSO-*d*_6_) δ 173.2, 170.6, 142.6, 129.7, 129.4, 128.1, 59.9, 51.4, 48.9, 33.8, 29.3, 28.6, 26.6, 24.7, 14.4. HRMS (ESI) *m*/*z*: [M + H]^+^ calcd. for C_23_H_31_N_2_O_3_ 383.2329; found 383.2327. HPLC–MS (gradient I) (M + H)^+^ = 383.2, *R*_t_ = 2.69 min (95%).

### General Procedure for the Synthesis of *N*′,*N*′-Diphenylamide Derivatives **44** and **45**

Diphenylamine (169.2 mg, 1.0 mmol) and DIPEA (0.418 mL, 3.0 mmol) were dissolved in ACN (9 mL). Then, the corresponding acyl chloride (1.2 mmol, indicated in each case) dissolved in ACN (3 mL) was added drop by drop at 0 °C. The solution was stirred at 0 °C during 30 min and overnight at room temperature. When the reaction had finalized (16 h), the mixture was diluted with EtOAc (20 mL) and washed with HCl 2 M (3 × 20 mL), NaHCO_3(sat)_ (3 × 20 mL), and NaCl_(sat)_ (3 × 20 mL). The organic phase was dried over MgSO_4_ anh., filtered, and evaporated under reduced pressure, obtaining a crude reaction that was purified in column chromatography eluting with the EtOAc/hexane solvent mixture (from 95:5 to 80:20).

#### *N*,*N*-Diphenylhexanamide (**44**)

Diphenylamine (169.2 mg, 1.0 mmol) and hexanoyl chloride (168 μL, 1.2 mmol) were used to yield **44** (202 mg, 76%) as a colorless oil. ^1^H NMR (300 MHz, DMSO-*d*_6_) δ 7.58–7.06 (m, 10H), 2.29–2.09 (m, 2H), 1.62–1.37 (m, 2H), 1.34–1.06 (m, 4H), 0.94–0.66 (m, 3H). ^13^C NMR (75 MHz, DMSO-*d*_6_) δ 172.3, 143.5, 129.7, 128.1, 34.7, 31.2, 24.9, 22.3, 14.2. HRMS (ESI) *m*/*z*: [M + H]^+^ calcd. for C_18_H_22_NO 268.1696; found 268.1688. HPLC–MS (gradient II) (M + H)^+^ = 268.2, *R*_t_ = 2.50 min (95%).

#### 2-(4-Bromophenyl)-*N*,*N*-diphenylacetamide (**45**)

Diphenylamine and 2-(4-bromophenyl)acetyl chloride (178 μL, 1.2 mmol) were used to yield **45** (260 mg, 71%) as a white solid; m.p. 90–92 °C (lit.^33^ 118–119 °C). ^1^H NMR (300 MHz, DMSO-*d*_6_) δ 7.45 (d, *J* = 8.4 Hz, 2H), 7.40 (m, 10H), 7.08 (d, *J* = 8.4 Hz, 2H), 3.56 (s, 2H). ^13^C NMR (75 MHz, DMSO-*d*_6_) δ 169.99, 143.13, 135.31, 131.92, 131.31, 129.38, 127.22, 119.98, 40.76. HRMS (ESI) *m*/*z*: [M + H]^+^ calcd. for C_20_H_17_BrNO 366.0488; found 366.0476. HPLC–MS (gradient II) (M + H)^+^ = 366.1, *R*_t_ = 2.49 min (95%).

### Antiviral Activity in an EBOV-GP-Pseudotyped Virus

#### Cell Lines

Human embryonic kidney cells 293T/17 (ATCC-CRL-11268) and human cervical adenocarcinoma cells HeLa (ATCC-CCL-2) were cultured in a Dulbecco modified Eagle medium (DMEM) at 37 °C and a 5% CO_2_ atmosphere, supplemented with 10% fetal bovine serum (FBS) and 1% l-glutamine.

#### Construction of an Ebola-GP-Y517S Mutant: Generation of Plasmid with Single-Point Mutation Y517S in Ebola Virus Glycoprotein

Plasmid encoding the Ebola-Makona virus glycoprotein mutation Y517S was carried out by following the Q5 Site-Directed Mutagenesis standard protocol (New England BioLabs).

Primer pairs containing the mutation Y517S of interest were designed using a New England BioLabs web-based design program (listed below):

EBO GP Y517S_F CAATTTACATTCCTGGACTACTCAGG
EBO GP Y517S_R GGGTTGCATTTGGGTTGA

Mutant construction was confirmed by sequencing, using an ABI PRISM 3100 genetic analyzer (Applied Biosystems) and posterior sequence analysis by Geneious R6 bioinformatics software. All the plasmids were prepared with HiPure Plasmid Filter Maxiprep (Invitrogen) and quantified by spectrophotometry (NanoDrop).

#### Production of Recombinant Viruses with Mayinga or VSV-G GP and an HIV Backbone

An EBOV-GP-pseudotyped lentiviral system was used to test the inhibitory activity of selected compounds. The viral construction was pseudotyped with the Zaire Ebola virus envelope glycoprotein (GP) strain Mayinga (GeneBank: U23187.1) or vesicular stomatitis virus envelope GP (VSV-G) and expressed luciferase as a reporter of the infection. For other experiments, Ebola Makona (GeneBank KM 233069.1) or Makona mutant Y517S was generated.

Recombinant viruses were produced in 293T cells by cotransfection with pNL4.3.Luc.R-E-plasmid (NIH AIDS Reagent Program, Division of AIDS: pNL4-3.Luc.R–.E– from Dr. Nathaniel Landau) and respective envelope glycoprotein using a standard calcium chloride transfection protocol (Life Technologies, Carlsbad, CA, USA).

Supernatants containing the recombinant viruses were harvested 48 h after transfection, centrifuged at 1200 rpm for 10 min at room temperature to remove cell debris, and stored frozen at −80 °C.

Infectious titers were estimated as tissue culture infectious dose per mL by limiting dilution (1:5 serial dilutions in triplicate) of the lentivirus-containing supernatants on HeLa cells. Luciferase activity was determined by a luciferase assay (Luciferase Assay System, Promega, Madison, WI).

#### Screening of Selected Compounds

All the compounds tested in this work were initially resuspended in DMSO at 1 mM.

Screening of selected compounds as EBOV-GP-pseudotype virus entry inhibitors was performed using HeLa cells (2 × 104 cells/well) in 96-well plates.

HeLa cells were incubated at 37 °C for 1 h with the compounds and then challenged with 5000 TCID (Tissue Culture Infective Dose) of recombinant viruses. After 48 h of incubation, cells were washed twice with PBS, lysed by addition of Steady-Glo Lyssis Buffer (Promega), and light measured in a GloMax-Multi+ detection system (Promega, Madison, WI, USA) with the luficerase assay system (Promega, Madison, WI).

Compounds that inhibited virus infection by more than 75% at a final concentration of 10 μM were further analyzed for potency, selectivity, and cytotoxicity. For these compounds, the range of concentrations tested was 10 nM–10 μM. As a control for selectivity, infection with VSV-G pseudoviruses was performed in the same conditions (Table S2 of the Supporting Information).

#### Toxicity Analysis of Compounds

HeLa (2 × 10^4^) cells were seeded in a 96-well plate and incubated with DMEM containing each compound at concentrations ranging from 0 to 200 μM. After 24 h, cell viability was measured by a colorimetric method based in the reduction to formazan of 3-(4,5-dimethylthiazol-2-yl)-5-(3-carboxymethoxyphenyl)-2-(4-sulfophenyl)-2*H*-tetrazolium (MTS) (Cell Titer 96 AQueous Nonradioactive Cell Proliferation Assay, Promega) following the manufacturer’s instructions.

Absorbance was recorded at 490 nm using an ELISA plate reader.

Cell viability was reported as the percentage of absorbance in treated cells relative to nontreated cells.

CC_50_ was calculated and nontoxic working concentrations (over 80% cell viability) used to test the activities of these compounds on EBOV-GP-pseudotyped lentiviral infection.

#### Statistical Analysis

The values of EC_50_ inhibition of the infection presented on the table correspond to the mean of three independent experiments. The EC_50_s values were estimated using GraphPad Prism v6.0 with a 95% confidence interval and settings for normalize dose–response curves.

#### Effect of the Ebola GP Y517S Mutation on the Inhibitory Capacity of Selected Compounds

293T cells were infected with Ebola wt pseudotypes or with the Y517S mutant in the presence of selected compounds (**11** or **13** at 2.5, 5 and 10 μM) previously incubated at 37 °C for 1 h with these cells. After 48 h, cells were lysed and light measured.

As control compounds, imipramine and toremifene at 1 μM were used.

### Antiviral Activity in the Wild-Type Zaire EBOV Mayinga Virus

Vero E6 cells (1 × 10^5^ cells per well in a 24-well plate) were infected with EBOV (Mayinga variant) at a multiplicity of infection (MOI) of 0.1. One hour later, the supernatant containing any unbound virus was discarded and the cells were washed once with 500 μL of PBS. During the incubation period, the compounds (initially resuspended in DMSO at 10 mM, were prepared and added to DMEM supplemented with 5% FBS and methyl cellulose. The concentration of the compounds ranged from 0.1 to 50 μM. After addition of the compounds to the cells, they were incubated at 5% CO_2_, 37 °C for 3 days. Concentration in the cell culture supernatant of infectious virus particles was then measured using an immunofocus assay as follows. The supernatant was discarded, and the cells were fixed in 4% paraformaldehyde for 1 h. The plates were washed thoroughly after fixation and in between each step thereafter. The cells were permeabilized with 0.5% Triton X-100 in PBS for 30 min followed by blocking with a solution of 5% FBS in PBS for 1 h. The primary antibody, polyclonal mouse anti-EBOV antibody (1:5000 in 2.5% blocking solution) was added followed by overnight incubation. The secondary antibody peroxidase-conjugated sheep anti-mouse IgG (H + L) (1:5000 in 5% blocking solution) was added followed by 1 h of incubation. To detect foci, tetramethylbenzidine (1:3 in distilled water) was added for 30 min or until spots developed after which it was discarded and the foci counted.

The concentrations that reduced virus titer by 50% (EC_50_) were calculated from dose–response curves using GraphPad Prism 9 with a 95% confidence interval.

### Cytotoxicity in Vero Cells

We pursued Vero cell viability and cytotoxicity tests of all reagents using the CellTiter 96 Nonradioactive Cell Proliferation Assay (Promega) following the Manufacturer’s instructions. We also studied the cytotoxic activity of the organic solvent DMSO. Based on these experiments, we selected optimal nontoxic working concentrations for infection assays. Stock solutions were dissolved in DMSO, and working solutions were freshly prepared in DMEM 2% fetal bovine serum (FBS) at the indicated concentrations. Incubation time was 24 h.

### Computational Protocol

The protonation state for **13** (see Figure S1 of the Supporting Information) was determined by using the pKa predictor of MarvinSketch (Marvin version 18.24, ChemAxon; https://www.chemaxon.com). The X-ray structure for the Zaire EBOV-GP in complex with toremifene with PDB ID 5JQ7(20) was used for docking and molecular dynamics (MD) simulations. The Glide docking software of the Schrödinger suite^23^ was used to obtain the binding mode for **13** into the proposed binding site between the GP1 and GP2 of the EBOV glycoprotein. Standard option joint with the SP algorithm was applied for pose generation and evaluation. The best ranked pose (see Table S1 of the Supporting Information) was considered for further explorations of the binding mode by means of MD simulations. Parameters for **13** in their docking-derived bioactive conformation were then calculated using the GAFF force field,^34^ and RESP^35^ charges were determined at the B3LYP/6-31G(d) level with Gaussian 09.^36^ The amber ffSB14 force field was used to describe the protein.^37^ Acetyl (ACE) and *N*-methyl (NME) capping groups were added to neutralize the N- and C-termini of the protein and a total of 15 disulfide bridges (C53_A-C_-C609_A-C_, C108_A-C_-C135_A-C_, C121_A-C_-C147_A-C_, C511_A-C_-C556_A-C_, C601_A-C_-C609_A-C_) were defined to assemble the fusion-competent homotrimer. The protein–ligand complex was then inserted in a truncated octahedron box with a layer of 20 Å, which was solvated and neutralized by adding 33,123 TIP3P^38^ water molecules and 3 Na^+^ ions for a total of 112,191 atoms. The system was energy minimized by applying 50,000 steps of the steepest descent algorithm followed by 5000 steps of the conjugate gradient algorithm. Equilibration was accomplished in six steps consisting on a gradual heating from 0 to 300 K in the NVT ensemble with Langevin dynamics^24^ by applying a collision frequency of 1 ps^–1^ followed by 5 ns of MD simulation in the NPT ensemble, which allowed to properly equilibrate the density prior to MD production. A distance restraint of 5 kcal mol^–1^ Å^–2^ was applied to some protein–ligand key interactions to avoid artificial distortions during the equilibration. This was gradually eliminated during the first 5 ns of MD production at constant volume and temperature (NVT), which were not considered in the final analysis of the MD trajectory. Accordingly, thee replicas of the protein–ligand complex were simulated for 300 ns at constant volume and temperature (NVT; 300 K) using periodic boundary conditions with Amber20^24^ for a total of 900 ns of MD production. The SHAKE method^39^ was applied to constraint bonds involving hydrogen atoms. A cutoff of 10 Å was used to treat short-range nonbonded interactions, and the particle mesh Ewald (PME) method was applied to manage long-range electrostatic interactions.^40^ A time step of 2 fs was used along the simulation. For the analysis, the *rms* and *atomfluct* commands of the CPPTRAJ20 module^41^ were used to evaluate the stability of the binding pose along the simulation, while the *distance* and *hbond* commands were used to evaluate the stability of relevant interactions established between **13** and the residues of the EBOV-GP fusion loop.

### EBOV-GP Production

A soluble EBOV-GP (Zaire strain Makona A82) was produced in mammalian cells. A cDNA coding for the GP (residues 32 to 311 and 463 to 634), with the mucin domain (312 to 462) replaced with the GSG sequence, was cloned in frame with the IgK leader sequence and an HA-tag (YPYDVPDYA) at the 5′ end and with a T4 fibritin trimerization sequence, a FLAG peptide and 6 × His-tag at the 3′ end, in the pcDNA3.1 vector. For expression, 10 cm dishes were seeded with 8 × 10^6^ HEK293T cells/dish and transfected with 20 μg/dish of the pcDNA/EBO-GP construct by the calcium phosphate method. About 3 days posttransfection, the EBOV-GP was purified by Ni-NTA affinity chromatography (Quiagen) from transfected cell supernatants, and it was transferred to 50 mM Tris pH 7.0 using a 100 kDa Amicon centrifugal filter unit. Protein concentration was determined with the Micro BCA protein assay kit (Thermo Scientific).

### STD–NMR Experiments

All NMR spectra were acquired at 298 K using a Bruker AVANCE 600 MHz spectrometer and processed with TOPSIN 4.1.4 software (Bruker, SA). NMR samples were prepared in deuterated Tris buffer 50 mM, NaCl 50 mM, pH 7.4.

STD–NMR experiments were performed with a 100:1 ligand-protein molar ratio and saturation time of 2 s. The protein was saturated in the aliphatic region of the spectrum at 0.8 ppm (on-resonance experiment), and the off-resonance experiment was saturated at 100 ppm.

Relative STD effects were calculated by comparing the intensity of the signals in the STD–NMR spectrum (ISTD) with signal intensities of the reference spectrum (off-resonance).

The STD signal with the highest intensity was set to 100%, and other STD signals were calculated accordingly. Control STD–NMR experiments were performed using an identical experimental setup and the same ligand concentration but in the absence of the protein.

Competition STD experiments were acquired with the same conditions as above. Increasing concentrations of imipramine were added in 1:1, 1:2, 1:3, and 1:4 ratios to **13**: with final concentrations of 300, 600, 900, and 1200 μM, respectively.

### Inhibitory Effect of Compounds on the EBOV-GPcl/NPC1-Domain C Interaction

Cleaved *EBOV-GP* (*EBOV-GPcl*) was generated *in vitro* using the bacterial protease thermolysin (250 μg/mL) (Sigma-Aldrich, St. Louis, MO) for 1 h at 37 °C and stopping the reaction by adding the metalloprotease inhibitor phosphoramidon (1 mM) (Sigma-Aldrich) for 20 min on ice.

#### NPC1-Domain C Construct (Plasmid)

A cassette vector based on *Homo sapiens* NPC1-mRNA NM-000271 encoding the following sequence elements was synthesized on a pcDNA3 plasmid: signal peptide (residues 1–24); domain C (residues 373–620); the first transmembrane domain (residues 267–295); Gly-Gly-Gly-Ser linker, and a triple Flag tag GeneArt (Thermo Fisher).

#### Expression, Purification, and Detection of NPC1-Domain C-Flag Fusion Protein

HEK293T cells (ATCC–CRL-11268) were transfected using Lipofectamine 3000 (Thermo Fisher) with the plasmid encoding NPC1-domain C-Flag. 36 h post transfection, cells were washed, lysed, and collected (Cell Lytic M-C2978, Sigma-Aldrich).

Proteins from the cell lysate were purified by affinity chromatography using an anti-Flag-M2 agarose column according to the manufacturer’s instructions (Sigma-Aldrich).

Detection of NPC1-domain C-Flag protein was performed by Western blot using an anti-Flag M2-peroxydase (1:1000) monoclonal antibody (Sigma-Aldrich).

#### EbolaGP-NPC1 Domain C Binding ELISAs

NPC1-domain C concentrations used in the ELISAs were normalized using a Micro BCA protein assay kit (Thermo).

Thermolysin-cleaved HIV-EBOV GP particles were captured onto high-binding 96-well ELISA plates (Corning, Corning, NY) using a conformation-specific anti-EBOV GP monoclonal antibody KZ52 (6.23 μg/mL).

Unbound viral particles were washed off, and purified Flag-tagged soluble NPC1-domain C (10 μg/mL) was added in the presence or not (control) of each compound (50 μM).

After that, bound flag-tagged proteins were detected with an anti-Flag antibody covalently conjugated to horseradish peroxidase (HRP) (1:5000) (Sigma-Aldrich). Finally, absorbance at 450 nm was measured after addition of the TMB substrate.

### Liver Microsome Stability Assay

Mouse or human liver microsomes and reduced nicotinamide adenine dinucleotide phosphate (NADPH) were obtained from Fisher Scientific SL. This assay gives information on the metabolic stability of early drug discovery compounds based on liver microsomes. Microsome stability was tested by incubating 10 μM of test compounds (**11** and **13**) and verapamil (as positive metabolized control) with 1.0 mg/mL hepatic microsomes (pooled human liver microsomes and pooled mouse (CD-1) liver microsomes) in 0.1 M potassium phosphate buffer (pH 7.4) with MgCl_2_ 5 mM. The reaction was initiated by adding NADPH (1 mM final concentration). Aliquots of 150 μL were collected at defined time points (0, 5, 15, 30, 45, and 60 min) and added to cold acetonitrile (150 μL) containing an internal standard (5 μg/mL warfarin) to stop the reaction and precipitate the protein. After stopping the reaction, the samples were centrifuged at 4 °C for 15 min and the loss of parent compounds was analyzed by HPLC–MS using single ion mode (SIM) detection. Data were log transformed and represented as half-life. All experiments were conducted by duplicate.

### Assessment of hERG Activity

The hERG potassium channel inhibition assay was carried out in hERG-expressed HEK293 cells using the FluxOR potassium assay and performed on a FLIPR TETRA (Molecular Devices) as outlined in the product information sheet from Invitrogen. As directed by the kit, the Powerload concentrate and water-soluble probenecid were added in the first step to enhance the dye solubility and retention, respectively. Then, FluxOR dye was added and mixed. The FluxOR loading buffer (NaCl 165 mM, KCl 4.5 mM, CaCl_2_ 2 mM, MgCl 1 mM, HEPES 10 mM, Glucose 10 mM) was adjusted to a pH of 7.2.

Media were removed from cell plates, and 50 μL of loading buffer containing the FluxOR dye mix was applied to each. The dye was removed after 60 min incubation at room temperature and the plates subsequently washed once with assay buffer, before adding the samples in assay buffer (a final volume of 50 μL). Plates were incubated 30 min at room temperature (25 °C) to allow equilibration of the test compounds. The thallium stimulation buffer (Tl_2_SO_4_ + K_2_SO_4_) was prepared according to the manufacturer’s instruction and injected into the plates on the FLIPR TETRA, to allow kinetic analysis from time zero (*t*_0_) to time 120 s (*t*_120_). Data obtained were analyzed using *Genedata Screener*.

The compounds were tested in triplicate using 10 points/1:2 dilution dose–response curves with the maximum concentrations at 50 μM. Astemizole was used as a positive control and DMSO 0.5% as a negative control.

### *In Vivo* Pharmacokinetic Studies

The study was conducted at AAALAC-accredited facility of Sai Life Sciences Limited, Hyderabad, India, in accordance with the Study Protocol SAIDMPK/PK-21-12-1218 and SAIDMPK/PK-21-06-582 for **11** and **13**, respectively. All procedures were in accordance with the guidelines provided by the Committee for the Purpose of Control and Supervision of Experiments on Animals (CPCSEA) as published in The Gazette of India, December 15, 1998. Prior approval of the Institutional Animal Ethics Committee (IAEC) was obtained before initiation of the study.

Healthy male BALB/c mice (8–12 weeks old) weighing between 20 and 35 g were used in the study. A total of 48 male mice were divided into two groups as group 1 (*n* = 24) and group 2 (*n* = 24) with a three mice per time point design. Animals in Group 1 were administered intraperitoneally (i.p.) with solution formulation of tested compound at 10 mg/kg dose. Animals in Group 2 were administered through oral route (p.o.) with solution formulation of tested compound at 50 mg/kg dose. In both cases, the formulation was based in 90% of phosphate buffer saline (PBS, pH 7.4) with a 5% solutol HS-15 and 5% *N*-methyl-2-pyrrolidone (NMP).

Blood samples (≈60 μL) were collected from a set of three mice at each time point (0.08 (for i.p. only), 0.25, 0.5, 1, 2, 4, 6 (for p.o. only), 8, and 24 h). In addition, along with terminal blood samples, brain samples were collected at 0.08 (for i.p. only), 0.25, 0.5, 1, 2, 4, 6 (for p.o. only), 8, and 24 h postdosing from three mice per time point. Immediately after blood collecting, brain samples were collected from a set of three animals for bioanalysis. Concentrations of the compound in mouse plasma and brain samples were determined by a fit-for-purpose LC–MS/MS method. The noncompartmental-analysis tool of Phoenix WinNonlin (ver. 8.0) was used to assess the pharmacokinetic parameters.

## Supporting Information Available

(Table S1) Docking with Glide of compound **13** into the proposed binding site between GP1 and GP2; (Table S2) antiviral activity of selected derivatives against VSV-G-pseudotype virus (pVSV-G); (Figure S1) distribution of microspecies as a function of pH for compound **13**; (Figure S2) root-mean-square-deviation (RMSD) analysis for the MD simulated EBOV-GP-**13** complex; (Figure S3) per-residue root-mean-square fluctuation (RMSF) for the protein backbone atoms of the MD simulated EBOV-GP-**13** complex; (Figure S4) chemical structures and related binding modes in the proposed binding site of the EBOV-GP for the antiviral compound toremifene, imipramine, **13**, and **11**; (Figure S5) superposition of the hit compound **1** (**SC816**) and compound **41** to the docking pose of compound **11** in the proposed binding site of the EBOV-GP; (Figure S6) decrease of the line broadening in the ^1^H spectrum of **13** due to the addition of imipramine; (Figures S7 and S8) STD experiments of compounds **11** and **41** in the presence of EBOV-GP protein; (Figure S9) HPLC chromatograms of lead compounds (**11** and **13**); and (Figures S10 and S11) ^1^H NMR and ^13^C NMR spectra of lead compounds (**11** and **13**) (PDF)
Molecular formula strings (CSV)

## Abbreviations

ACN: acetonitrile
ADME: absorption, distribution, metabolism, and excretion
AUC_last_: area under the plasma concentration–time curve from time zero to the time of the last quantifiable concentration
BSL-4: biosafety level 4
B3LYP: Becke, 3-parameter, Lee–Yang–Parr
CL_int_: intrinsic clearance
CC_50_: 50% cytotoxic concentration
*C*_max_: peak serum concentration
DIPEA: *N*,*N*-diisopropylethylamine
DMSO: dimethyl sulfoxide
EBOV: Ebola Virus
EBOV-GP: Ebola Virus glycoprotein
EBOV May: Zaire EBOV Mayinga 1976 strain
EC_50_: 50% effective concentration
EVD: Ebola Virus disease
FDA: Food and Drug Administration
GAFF: general amber force field
GP: glycoprotein
GPcl: cleaved glycoprotein
HB: hydrogen bond
hERG: human ether-a-go-go related gene
IC_50_: 50% inhibitory concentration
i.p.: intraperitoneal
Kp: brain/plasma ratio
L: RNA-dependent RNA polymerase
SAR: structure–activity relationships
SI: selectivity index
MD: molecular dynamics
NME: *N*-methyl
NP: nucleoprotein
NPC1: Niemann–Pick C1 protein
NVT: canonical ensemble with constant number of particles, volume, and temperature
pEBOV: EBOV-GP-pseudotyped virus
PK: pharmacokinetic
PME: particle mesh Ewald
p.o.: oral
PS: π-stacking
RESP: restrained electrostatic potential
RMSD: root-mean-square deviation
RMSF: root-mean-square fluctuation
rVSV: recombinant vesicular stomatitis virus
rVSV-EBOV-GP: recombinant cesicular stomatitis virus-Ebola virus-glycoprotein
STD–NMR: saturation transfer difference–nuclear magnetic resonance
THF: tetrahydrofuran
TIP3P: transferable intermolecular potential with 3 Point
*T*_max_: time to reach *C*_max_
VP 24, 30, 35, and 40: viral protein 24, 30, 35, and 40
VSV: vesicular stomatitis virus
VSV-G: vesicular stomatitis virus envelope GP
