# Supplementary material for: N′-Phenylacetohydrazide Derivatives as Potent Ebola Virus Entry Inhibitors with an Improved Pharmacokinetic Profile
Source: J Med Chem. 2023 Apr 6;66(8):5465–83. doi: 10.1021/acs.jmedchem.2c01785 (PMC10150359; doi:10.1021/acs.jmedchem.2c01785)
Supplement: Supplementary file 1 — jm2c01785_si_001.pdf [file jm2c01785_si_001.pdf]

SUPPORTING INFORMATION

N'-phenylacetohydrazide derivatives as potent Ebola virus entry inhibitors with improved pharmacokinetic profile

Alfonso Garcia-Rubia,[1,†] Fátima Lasala,[2, †] Tiziana Ginex,[1] Marcos Morales-Tenorio,[1] Catherine Olal,[3] Michelle Heung,[3] Paola Oquist,[4] Inmaculada Galindo,[5] Miguel Ángel Cuesta-Geijo,[5] José M. Casasnovas,[6] Nuria E. Campillo,[1,7] Ángeles Canales,[4] Covadonga Alonso,[5] Ana Martínez,[1] César Muñoz-Fontela,[3] Rafael Delgado,[2,*] Carmen Gil[1,*]

[1]Centro de Investigaciones Biológicas Margarita Salas (CIB-CSIC), 28040 Madrid, Spain

[2]Instituto de Investigación Hospital 12 de Octubre, 28041 Madrid, Spain

[3]Bernhard Nocht Institute for Tropical Medicine, 20359 Hamburg, Germany

[4]Facultad de Ciencias Químicas, Universidad Complutense de Madrid, 28040 Madrid, Spain

[5]Dpt. Biotechnology, Instituto Nacional de Investigación y Tecnología Agraria y Alimentaria (INIA-CSIC), 28040 Madrid, Spain

[6]Centro Nacional de Biotecnología (CNB-CSIC), 28049 Madrid, Spain

[7]Instituto de Ciencias Matemáticas (ICMAT-CSIC), 28049 Madrid, Spain

[†]These authors have contributed equally to this work

* Correspondence: rafael.delgado@salud.madrid.org (R. D.), carmen.gil@csic.es (C. G.)

Table of content

TABLES

Table S1. Docking with Glide of compound **13** into the proposed binding site between GP1 and GP2.

Table S2. Antiviral activity of selected derivatives against VSV-G-pseudotype virus (pVSV-G).

FIGURES

Figure S1. Distribution of microspecies as a function of pH for compound **13**.

Figure S2. Root mean-square deviation (RMSD) analysis for the MD simulated EBOV-GP-**13** complex.

Figure S3. (A) Per-residue root mean-square fluctuation (RMSF) for the protein backbone atoms of the MD simulated EBOV-GP-**13** complex.

Figure S4. Chemical structures and related binding modes in the proposed binding site of the EBOV-GP for the antiviral compound toremifene (**A**), imipramine (**B**), **13** (**C**), and **11** (**D**).

Figure S5. Superposition of the hit compound **1** (**SC816**) and compound **41** to the docking pose of compound **11** in the proposed binding site of the EBOV-GP.

Figure S6. Decrease of the line broadening in the ^{1}H spectrum of **13** due to the addition of imipramine.

Figure S7 and **S8**. STD experiments of compounds **11** and **41** in the presence of EBOV-GP protein

Figure S9. HPLC chromatograms of lead compounds (**11** and **13**)

Figure S10 and **S11**. ^{1}H NMR and ^{13}C NMR spectra of lead compounds (**11** and **13**)

Table S1. Docking with Glide of compound **13** into the proposed binding site between GP1 and GP2 of the EBOV-GP.

Rank	Cluster	Cmp	GScore (kcal/mol)
**1**	**1**	**13**	**-7.91**
2	1	13	-7.76
3	1	13	-7.60
4	1	13	-7.53
5	1	13	-7.50

Table S2. Antiviral activity of selected derivatives against VSV-G-pseudotype virus (pVSV-G).

	%inh@10 µM (pVSV-G)
**1**	25%@10 µM
**2**	10%@10 µM
**3**	10%@10 µM
**6**	9%@10 µM
**7**	30%@10 µM
**9**	-10%@10 µM
**10**	2%@10 µM
**11**	50%@10 µM
**12**	-29%@10 µM
**13**	42%@10 µM
**14**	2%@10 µM
**30**	-25%@10 µM
**32**	-36%@10 µM

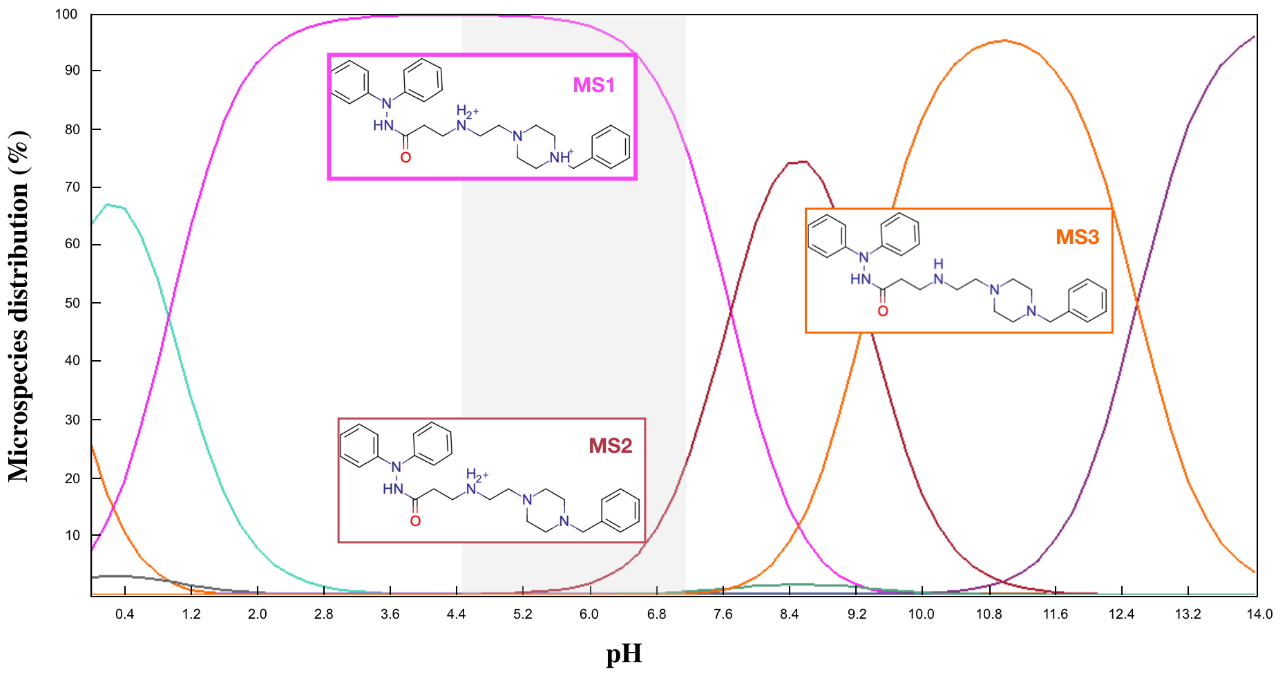

Figure S1. Distribution of microspecies as a function of pH for compound **13**. For clarity, only the chemical structure for the most relevant microspecies (MS1-3) is shown.

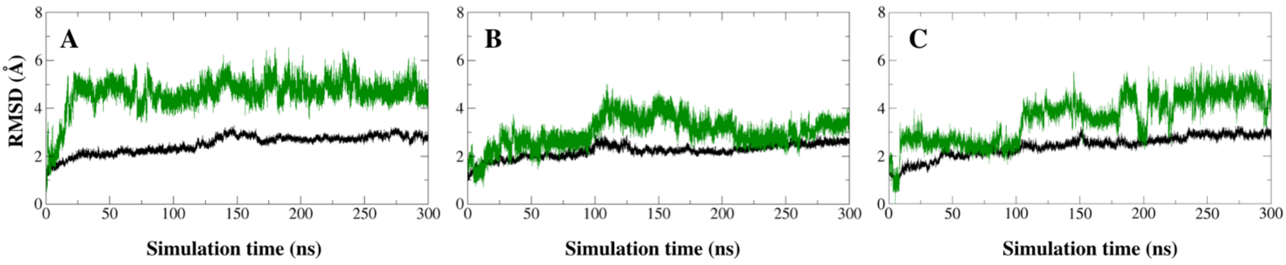

Figure S2. Root mean-square deviation (RMSD) analysis for the MD simulated EBOV-GP-**13** complex. Time-course profiles for the protein backbone atoms and for the compound **13** are reported respectively in black and green. For statistical consistency, simulations were done in triplicate.

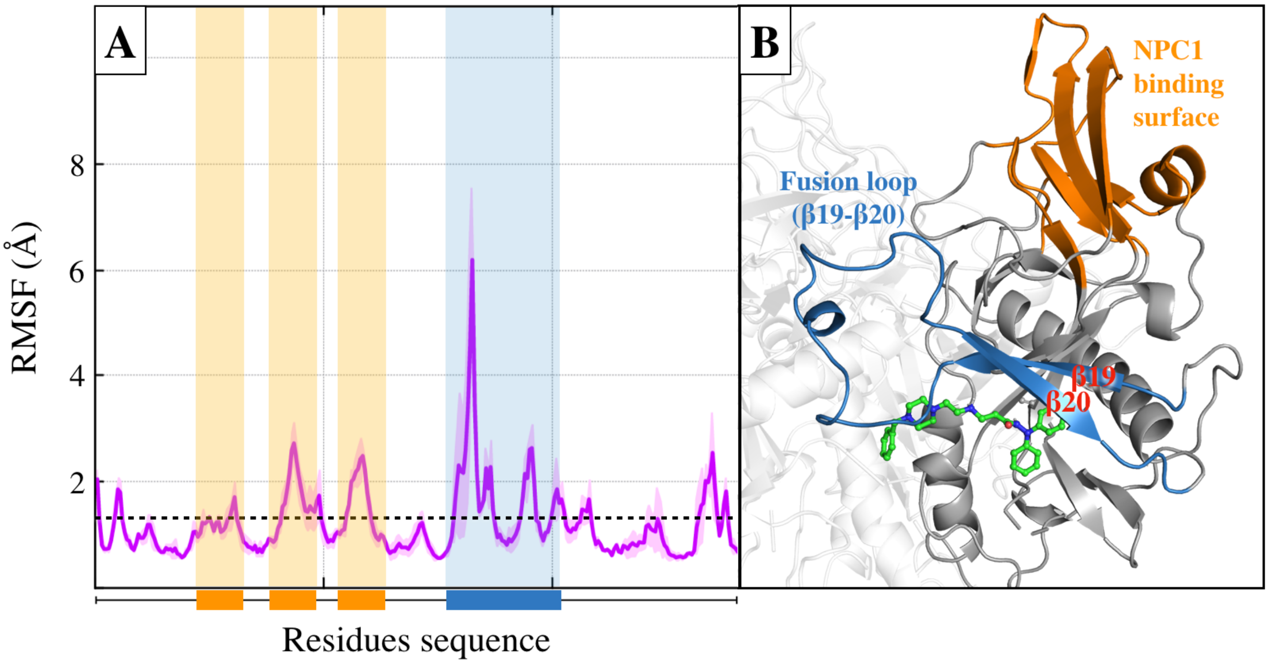

Figure S3. (**A**) Per-residue root mean-square fluctuation (RMSF) for the protein backbone atoms of the MD simulated EBOV-GP-**13** complex expressed as mean (pink line) and standard deviation (pink shading) along the three simulated replicas. Black dotted line marks the mean RMSF value along all the residues. (**B**) Residues forming the NPC1 binding surface and the fusion loop (β19- β20) of the EBOV-GP are highlighted respectively in orange and blue. Compound **13** is shown in green sticks.

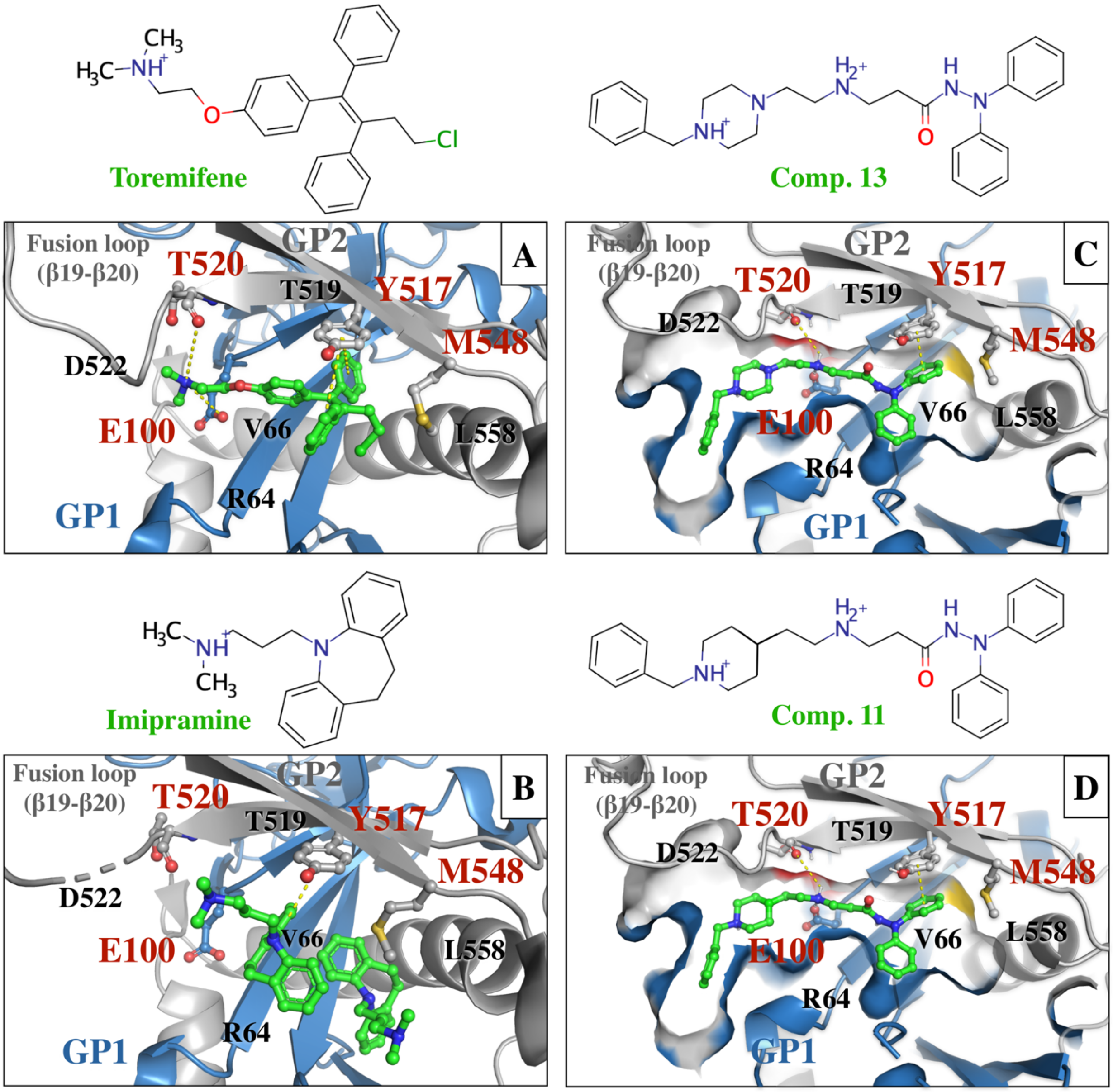

Figure S4. Chemical structures and related binding modes in the proposed binding site of the EBOV-GP for the antiviral compound toremifene (**A**), imipramine (**B**), **13** (**C**), and **11** (**D**). Residues $R64_{GP1}$, $V66_{GP1}$, $E100_{GP1}$, $Y517_{GP2}$, $T519_{GP2}$, $T520_{GP2}$, $D522_{GP2}$ and $L558_{GP2}$ shaping the crystallographic binding site are shown. Among them, those involved in relevant protein-ligand interactions (red label) are highlighted.

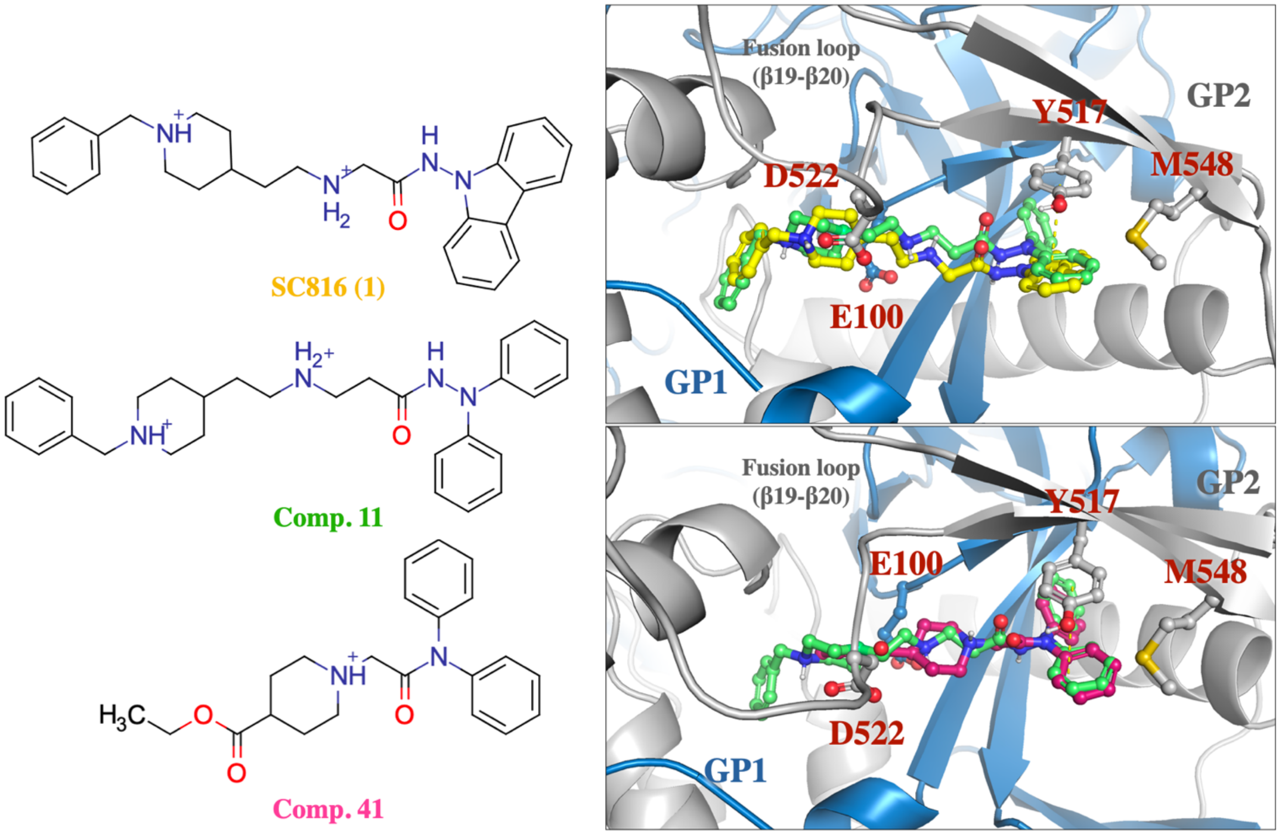

Figure S5. Superposition of the hit compound **1** (**SC816**; in yellow) and compound **41** (in magenta) to the docking pose of compound **11** (in green) in the proposed binding site of the EBOV-GP. Residues mainly involved in protein ligand interactions are highlighted in red.

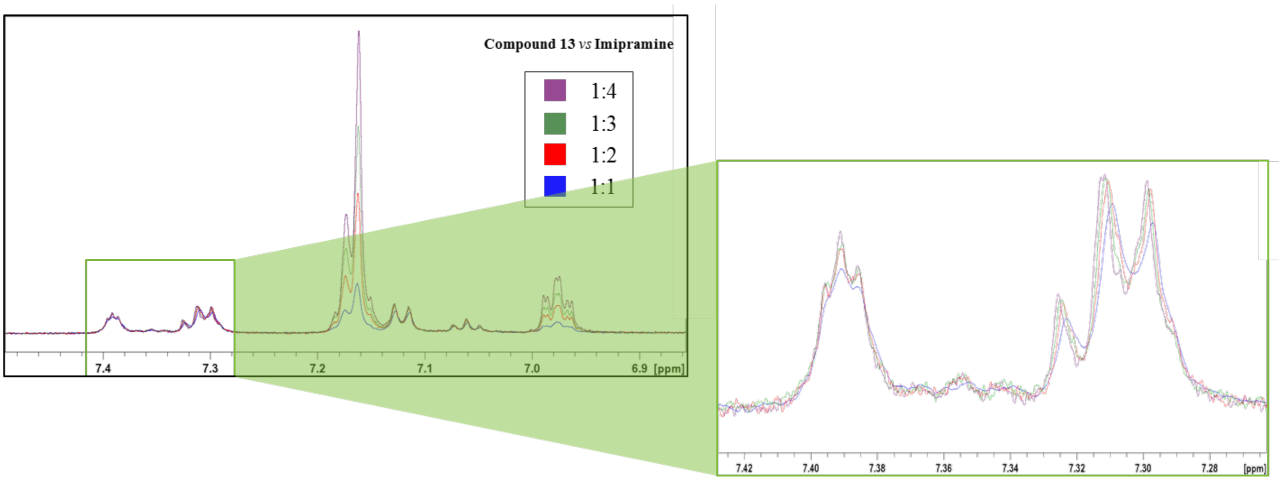

Figure S6. Decrease of the line broadening in the ^{1}H spectrum of **13** due to the addition of imipramine.

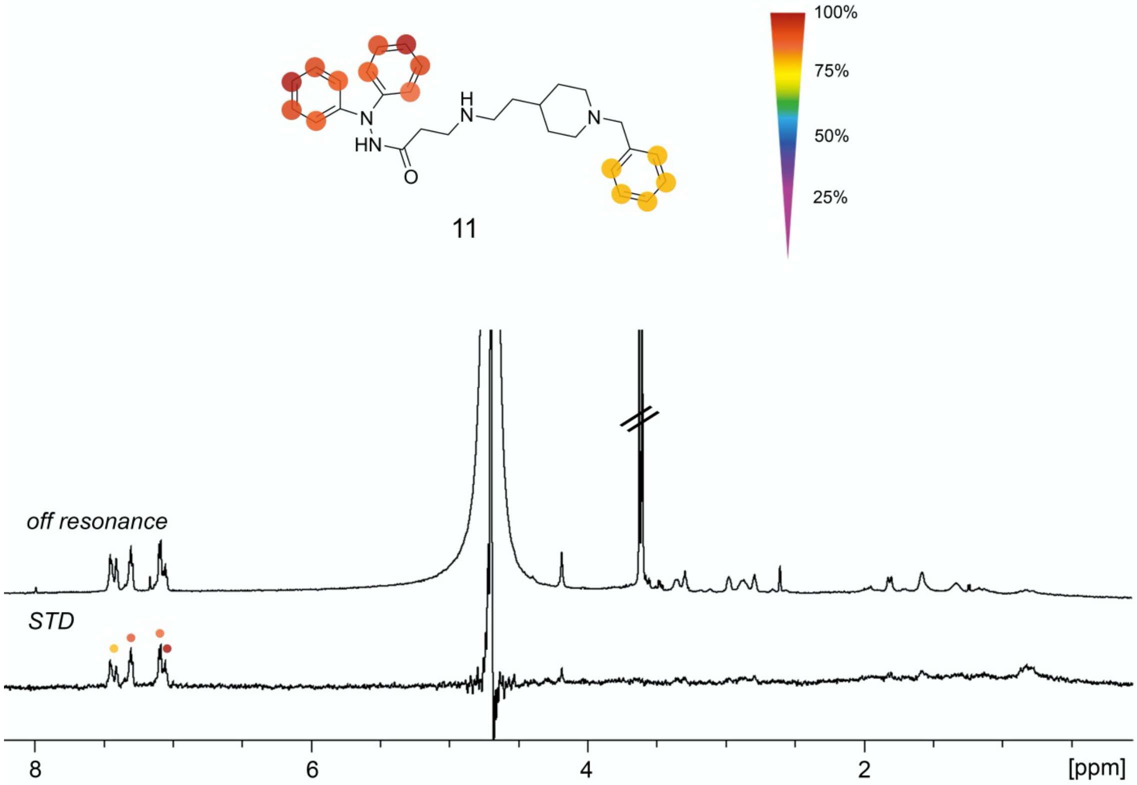

Figure S7. STD NMR experiment of compound **11** acquired in the presence of EBOV-GP protein. Off resonance spectrum of the same sample. The buffer signal is labelled with black lines. Mapping of the STD effects on the structure of compound **11**.

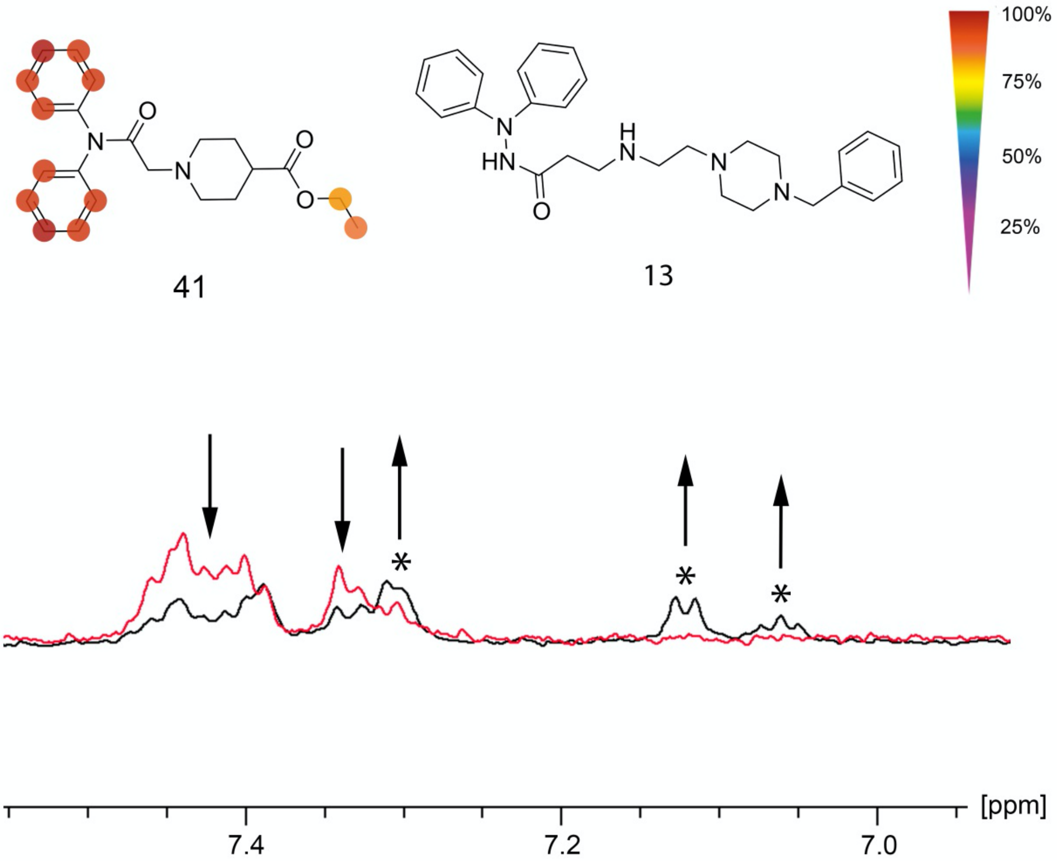

Figure S8. Red color, STD NMR experiment of compound **41** acquired in the presence of EBOV-GP protein. Black color, STD NMR experiment of **41** in the presence of EBOV-GP protein upon addition of **13**. The arrows highlight the decrease of the STD signals of **41** and the appearance of the signals corresponding to compound **13**.

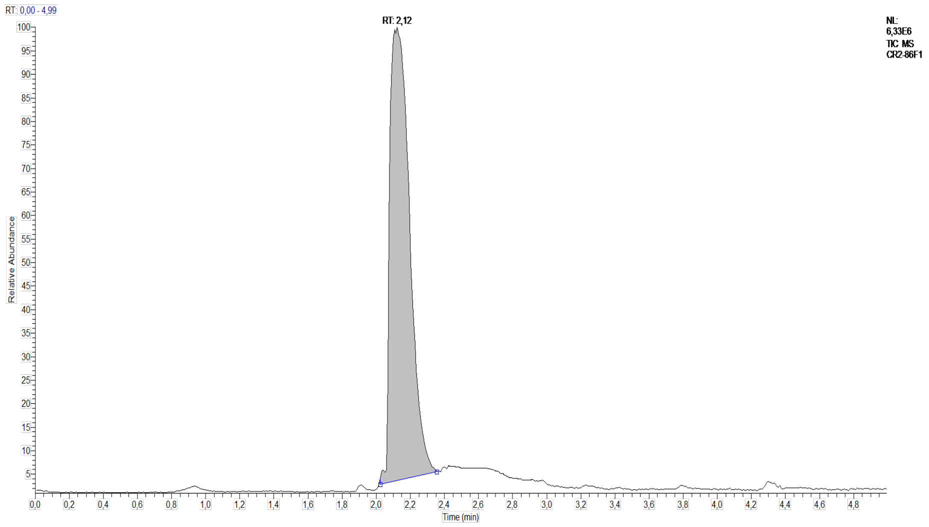

Compound **11**

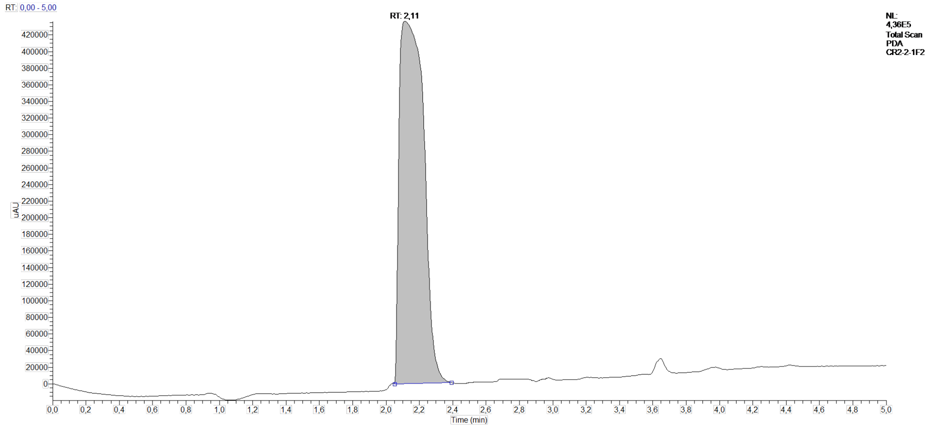

Compound **13**

Figure S9. HPLC chromatograms of lead compounds (**11** and **13**).

Compound **11**

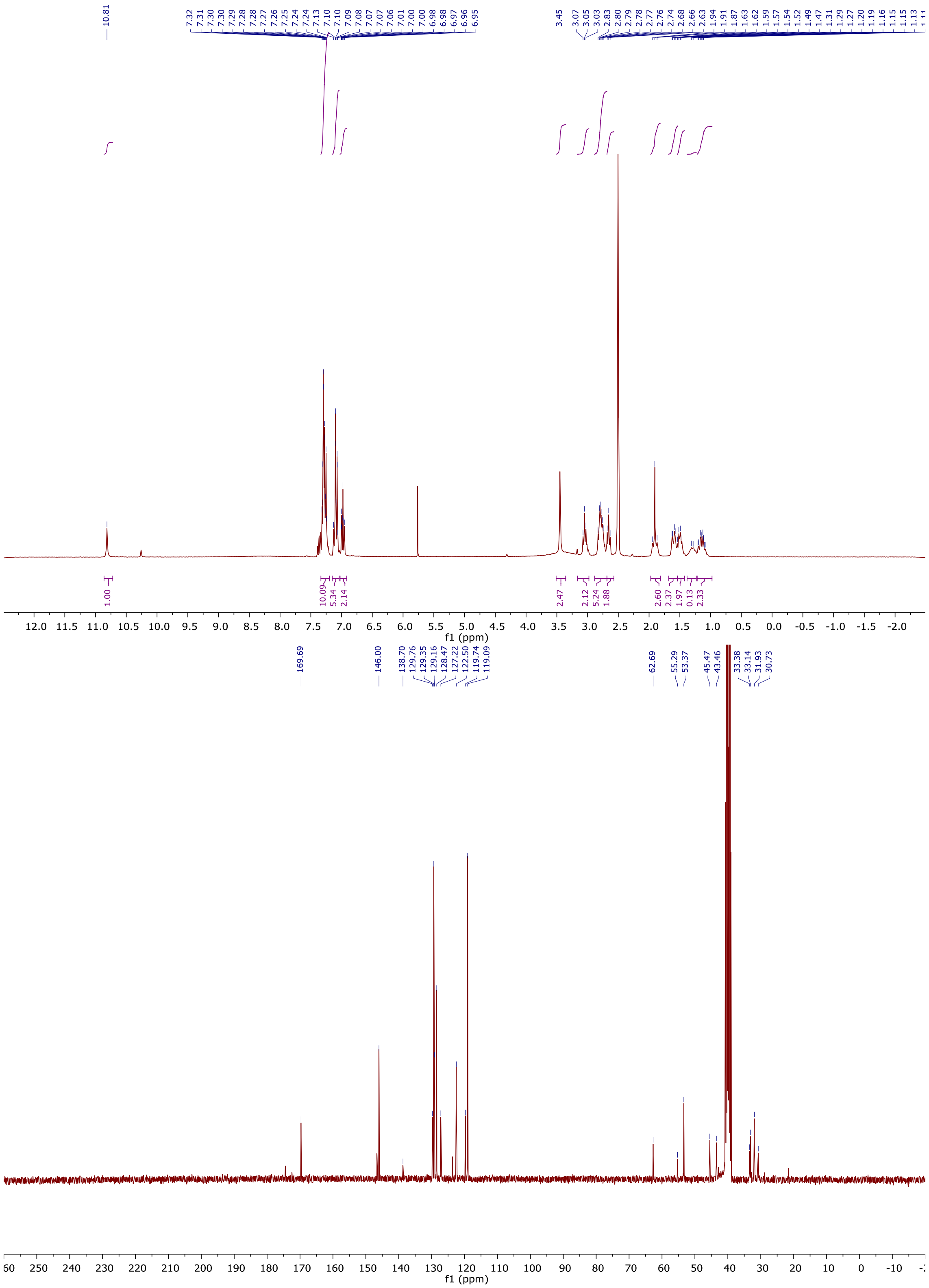

Figure S10. ^{1}H NMR and ^{13}C NMR spectra of lead compound **11**.

Compound **13**

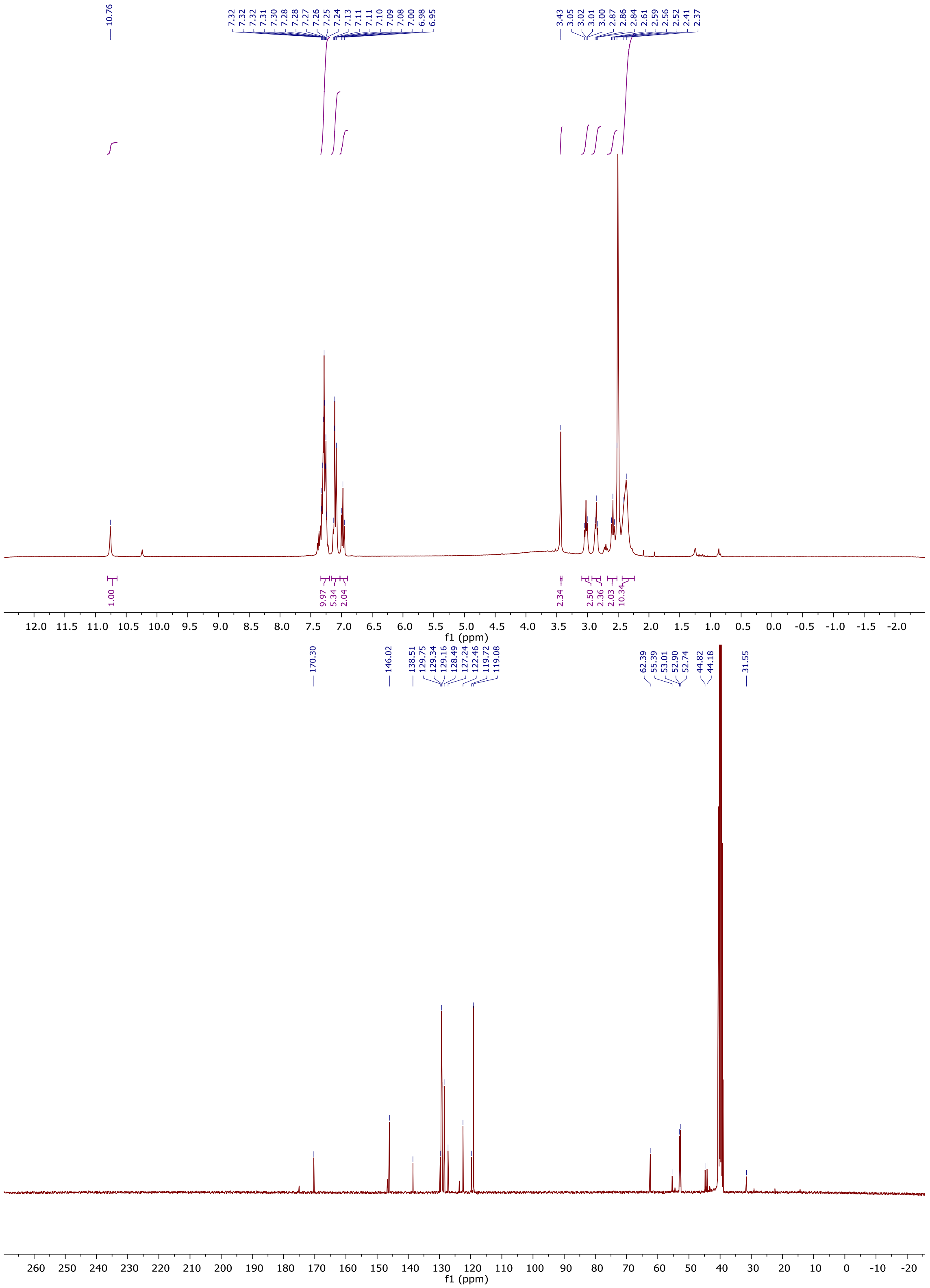

Figure S11. ^{1}H NMR and ^{13}C NMR spectra of lead compound **13.**
